# Budburst and Flowering Dynamics of Cultivated Olive Across the Mediterranean Basin

**DOI:** 10.3390/plants15192980

**Published:** 2026-09-29

**Authors:** Rüştü Efe Değer, Hristofor Miho, Esteban Meca, Diego Cabello Pozo, Georgios Koubouris, Ioanna Manolikaki, Sara Oulbi, Omar Abou-Saaid, Enzo Perri, Samanta Zelasco, Hülya Kaya, Songül Acar, Concepción Muñoz Díez

**Affiliations:** 1 Department of Agronomy, Universidad de Córdoba (UCO), Campus de Rabanales, 14071 Córdoba, Spain; rustuefe.deger@gmail.com (R.E.D.); hmiho@uco.es (H.M.);; 2 Department of Applied Physics, Radiology and Physical Medicine, Universidad de Córdoba (UCO), Campus de Rabanales, 14071 Córdoba, Spain; 3 Institute of Olive Tree, Subtropical Crops and Viticulture, Hellenic Agricultural Organization-DIMITRA, Leoforos Karamanli 167, GR-73134 Chania, Greece; 4 Regional Center of Agricultural Research of Marrakech, Institut National de la Recherche Agronomique (INRA), Les Portes de Marrakech, Marrakech P.O. Box 533, Morocco; 5 Research Centre for Olive, Fruit and Citrus Crops (CREA-OFA), Council for Agricultural Research and Economics (CREA), Contrada Li Rocchi Vermicelli 83, 87036 Rende, Italy; 6 Department of Breeding and Genetics, Olive Research Institute, 35100 İzmir, Türkiye

**Keywords:** *Olea europaea*, phenology, germplasm bank, budburst, flowering, chilling accumulation, forcing, phenological dispersion, Mediterranean climate, climate adaptation

## Abstract

Olive reproductive phenology depends on the balance between winter chilling and subsequent heat accumulation, yet multi-environment Mediterranean observations to assess this dynamic across thermal regimes remain scarce. We monitored Full Budburst (FB) and Full Flowering (FF) during 2021–2024 in 670 cultivars across five germplasm banks in Greece, Italy, Morocco, Spain, and Türkiye. Median dates, periods, dispersion, and phenological stage overlap were related to bioclimatic factors and chilling and heat accumulation. The influence of the germplasm-bank environment, year, and their interaction on the timing of both stages was assessed. The Median Full Budburst (FB_50_) and Median Full Flowering date (FF_50_) were strongly correlated. Likewise, budburst dispersion (ID*_bb_*) was positively associated with flowering dispersion (ID*_ff_*), serving as descriptive indicators of phenological synchrony under varying thermal regimes. Warmer conditions between FB_50_ and FF_50_ were associated with a shorter transition to flowering. Conversely, warmer pre-budburst conditions and lower chilling accumulation were associated with greater budburst and flowering dispersion and reduced synchrony. These findings indicate that budburst provides an important temporal reference for flowering. Integrating budburst and flowering dispersion with transition gap (T*_gap_*) metrics offers a quantitative framework for characterizing population-level phenological responses under contrasting thermal regimes and enhancing future olive breeding programs.

## 1. Introduction

The olive tree (*Olea europaea* L.) is the most extensively cultivated woody fruit crop worldwide, covering more than 11 million hectares, with over 90% concentrated in the Mediterranean Basin [1]. Olive cultivation relies on hundreds of traditional cultivars highly adapted to local edaphoclimatic conditions [2]. However, crop stability is currently threatened by two concurrent factors: climate change, which is altering temperature and rainfall patterns across historical growing regions, and the expansion of olive orchards into non-Mediterranean zones such as Brazil or China, where climatic adaptation remains uncertain [3,4].

The precise synchronization of the phenological cycle reflects overall crop adaptation to local environmental conditions [4,5]. This alignment is critical for perennial woody trees, which cannot be replaced on an annual basis. Within this cycle, flowering represents a vital stage due to its direct link with the fruit set and final yield. Global warming is severely threatening this equilibrium, particularly in species like the olive, where winter cold temperatures act as the primary floral inductor [6,7]. Olive buds enter winter morphologically undifferentiated, and exposure to a specific chilling temperature range determines whether they commit to reproductive development or remain vegetative. Although the existence of true endodormancy in olives remains under debate [8], once chilling requirements are satisfied, a distinct phase of heat accumulation (forcing temperatures) is necessary to break ecodormancy and initiate budburst; from this point onward, a thermal integral regulates the onset of flowering [6,7]. Consequently, sudden thermal fluctuations and unseasonal heat spikes frequently destabilize the cycle, resulting in asynchronous development and erratic flowering fluxes [9,10].

In this context, budburst represents a critical stage preceding flowering. Since it occurs chronologically closer to floral induction and ecodormancy release, its progress may serve as a more direct physiological indicator of the fulfilment of the temperature conditions associated with these processes than flowering [4,7]. Furthermore, not only the timing of budburst and flowering, but also the time to complete these processes, might be indicative of cultivar adaptation [4]. Asynchronous budburst and extended flowering periods often reflect insufficient winter chilling, contributing to erratic fruit set, uneven fruit ripening, and significant reductions in the oil yield and quality [4,10]. Although critical for climate adaptation, the duration of both phases remains understudied, while for budburst, neither its progression dynamics nor its duration have been incorporated into olive phenological research and modeling.

Overall, olive phenological data remain scarce and predominantly focused on flowering dates, while being geographically fragmented and recorded using inconsistent methodologies [4,11,12,13]. Consequently, a unified, basin-wide perspective on olive budburst and flowering dynamics across the Mediterranean Basin does not yet exist. Establishing this macroecological framework is crucial, not only for evaluating current and future climate change impacts on the olive distribution and yield potential, but also for addressing public health concerns given the high allergenicity of olive pollen [14,15]. International germplasm banks are uniquely positioned to resolve these limitations, by maintaining hundreds of cultivars in live field collections. These repositories function as representative populations that capture the broad genetic diversity and environmental plasticity of the crop [2,16].

Coordinated monitoring across international repositories permits consistent comparisons of reproductive phenology across contrasting environments and broad genetic resources. Such networks can support the evaluation of chilling–forcing relationships and modeling and provide a basis for future multi-environment breeding studies [17,18].

Modifying the flowering date has long been a classic target in fruit breeding [19]. Selecting late-flowering cultivars has expanded the cultivation of species like almond into areas prone to late spring frosts during flowering [20,21,22]. Conversely, targeting early flowering can be advantageous in other situations; for instance, in species such as apricot or peach, early flowering is linked to lower chilling requirements, making these genotypes highly valuable for adaptation to warmer winters [23,24,25]. In olives, the flowering date is also becoming a key breeding objective [16,26]. However, while species such as apple, almond, or peach show remarkable variability with flowering dates spanning several weeks [21,27], flowering in olive trees appears to be highly synchronous. Long-term datasets from the international collections of Córdoba (Spain) and Marrakech (Morocco), which preserve hundreds of cultivars, reveal that most accessions reach Full Flowering within a window of just one week [11,16,26]. This synchronized flowering might be an adaptive response to ensure pollination in an anemophilous, highly self-incompatible species [28]. However, if confirmed, this synchronicity poses a potential barrier for crop breeding.

To address this question, we adopted a population-level approach to assess olive reproductive phenology across the Mediterranean basin. Rather than examining isolated cultivars, this study coordinates the evaluation of budburst and flowering across five major germplasm banks over multiple years, quantifying their timing, dispersion, and overlap across diverse thermal environments. We monitored budburst and flowering from 2021 to 2024 across five international germplasm banks: WOGB-UCO (Córdoba, Spain), INRA (Marrakech, Morocco), ELGO-DEMETER (Chania, Greece), CREA (Mirto Crosia, Italy), and ORI (İzmir, Türkiye). These repositories span a broad climatic and microclimatic gradient across the Mediterranean Basin, ranging from semi-arid inland conditions in Marrakech and continental Mediterranean conditions in Córdoba to coastal and insular Mediterranean microclimates in Chania, Mirto Crosia, and İzmir. All collections were evaluated using shared phenological descriptors, and monitoring protocols developed within the Gen4olive H2020 project. We hypothesized that collections exposed to warmer winter conditions would exhibit greater phenological dispersion and an extended budburst duration due to incomplete chilling accumulation, whereas cooler sites with adequate chilling would display higher reproductive synchrony. To test this hypothesis, our objectives were as follows: (i) to characterize the geographical and interannual dynamics of budburst and flowering across the Mediterranean Basin; (ii) to quantify the collection-level duration, dispersion, and overlap of both phenological stages; and (iii) to examine their relationships with key climatic variables, including winter chilling accumulation and temperatures.

## 2. Results

### 2.1. Bioclimatic Diversity of the Germplasm Banks

We monitored olive phenology in five GBs across the Mediterranean Basin. The WBCS indices structured the five GBs into two primary bioclimatic groups (Table S1). The first group comprised the oceanically influenced sites: Morocco, Greece, and Italy. These locations are characterized by low continentality index values (*I_c_* < 19), high annual mean temperatures (*T_ann_* > 17 °C), and elevated thermicity index values (*I_tc_* > 350), reflecting mild winter conditions, with Morocco representing the most arid and thermo-extreme boundary (*P_ann_* = 308.2 mm). The second group clustered Spain and Türkiye under a well-defined pluviseasonal continental pattern. This cluster was characterized by the highest continentality indices in the dataset (*I_c_* > 21.5) and more moderate annual mean temperatures (~16 °C), supporting their geographic segregation from the oceanic influence.

This substantial environmental diversity was confirmed by the Principal Component Analysis (PCA) performed on the 19 standard bioclimatic variables (BIO 1 to BIO 19) (Figure S2; Table S2). The first two principal components explained 64.9% of the total variance. PC1 accounted for 39.0% of the variance and captured a clear gradient of continentality and aridity versus maritime buffering and the precipitation level, which separated the collections of Spain, Türkiye, and Morocco from the oceanic regimes of Italy and Greece (BIO 2, 3, 5, and 12). PC2 explained 25.9% of the variance and captured winter mildness (BIO 1, 4, 6, and 11), effectively separating the warmer, low-latitude collections of Morocco and Greece from the cooler collections located at higher latitudes in Spain, Italy, and Türkiye (Table S2).

Taken together, these environmental analyses grouped the studied sites into distinct climatic profiles: Greece and Italy are defined by buffered maritime conditions, Morocco represents the arid extreme, and Spain and Türkiye exhibit marked continentality. In terms of thermal regimes, Morocco emerged as the warmest location, whereas Türkiye was the coldest.

### 2.2. Fixed and Dynamic Temperature Windows During the Evaluated Period (2021–2024)

We calculated fixed-seasonal and phase-specific temperature windows to assess their relationships with olive phenology across the five GBs during the study period.

The fixed seasonal temperature window extended from 1 February to 31 May (*T_mean_*_,*Feb-May*_); it increased progressively during the evaluated period across the network, rising from 14.52 °C in 2021 to 15.89 °C in 2024 (Figures S3 and S4). Morocco was consistently the warmest site (*T_Feb-May_* 4-year mean: 18.27 °C) peaking at 19.09 °C in 2023, with average maximum daily temperatures reaching 27.49 °C. In contrast, Türkiye was the coldest site (*T_Feb-May_* 4-year mean: 12.50 °C) dropping to a minimum annual mean of 11.75 °C in 2023.

Furthermore, a marked contrast in daily temperature oscillations was observed between sites. Continental collections, Spain and Türkiye, along with Morocco, exhibited large daily thermal ranges of 12.0 °C to 15.0 °C during this February–May period. Conversely, the maritime influenced GBs (Greece and Italy) displayed highly buffered thermal regimes with much narrower daily ranges (e.g., Greece showing a remarkably reduced mean daily thermal range of 5.09 °C).

**Figure 1:**
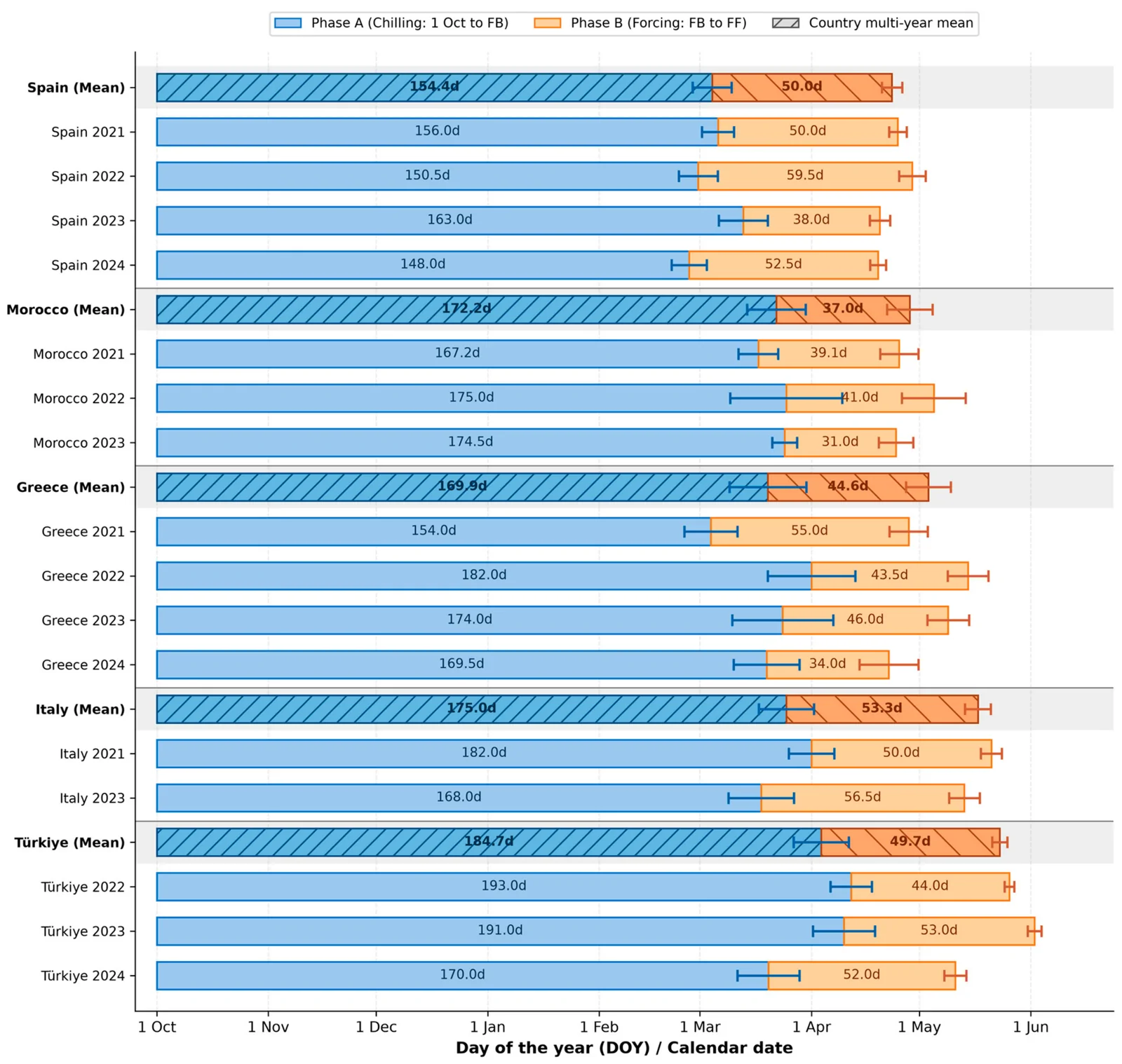
Timing and duration of Phase A and Phase B across Mediterranean germplasm-bank environments and years. Note: Phase A (blue) extends from 1 October to *FB*_50_ and Phase B (orange) from *FB*_50_ to *FF*_50_. Numbers indicate the duration in days, hatched rows show country means, and error bars represent the collection-level standard deviation around the median dates. *FB*_50_ and *FF*_50_, Median Full Budburst and Full Flowering dates.

**Figure 2:**
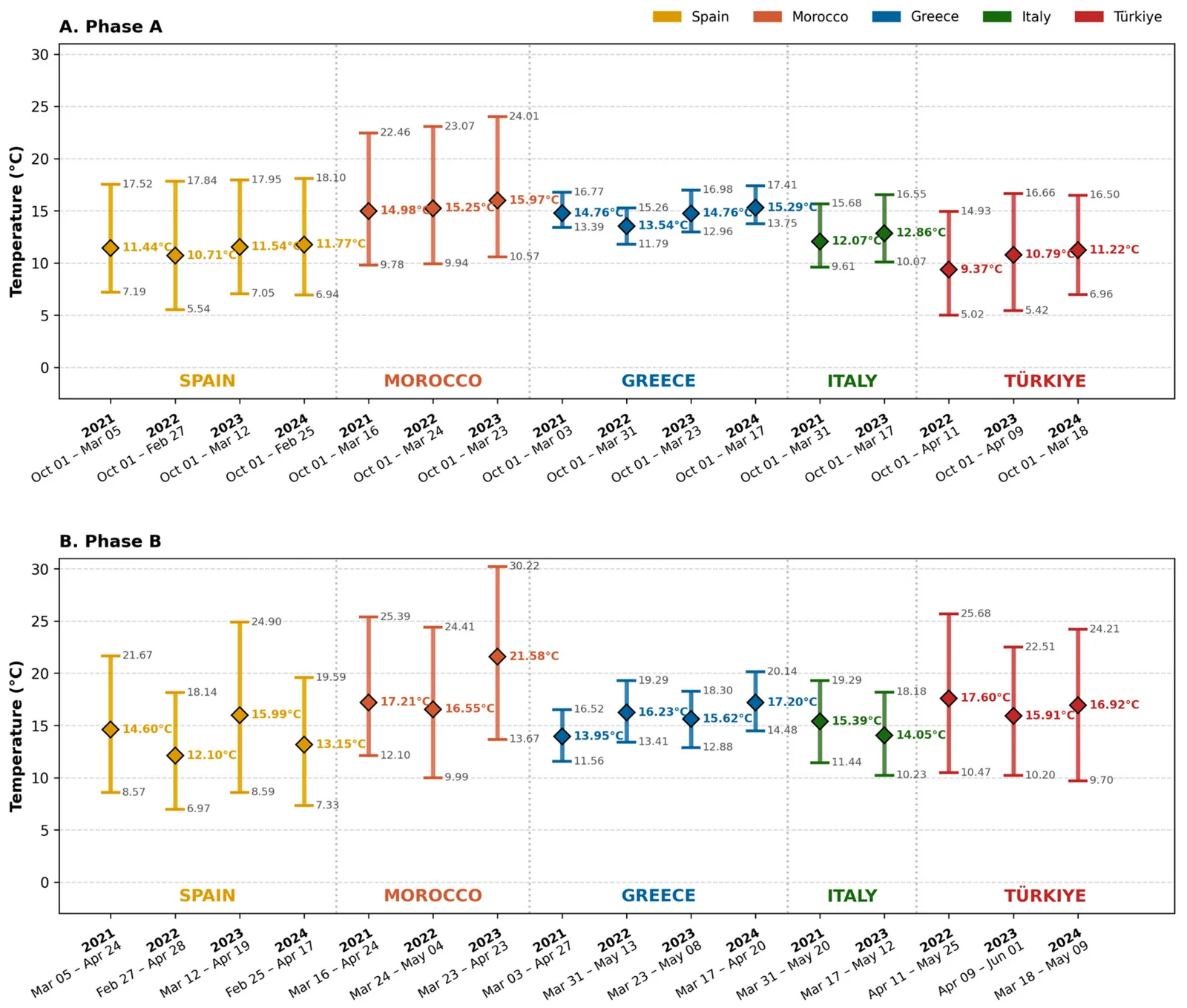
Phase-specific temperature conditions across Mediterranean germplasm-bank environments and years. Note: (**A**) Phase A, from 1 October to *FB*_50_; (**B**) Phase B, from *FB*_50_ to *FF*_50_. Diamonds show the mean temperature and vertical bars the minimum–maximum daily temperature range.

We also calculated temperatures within two phase-specific windows (Phase A and Phase B) for each site-year as developmental indicators:

Phase A—from 1 October to Full Budburst, *FB*_50_: mean duration of Phase A ranged from 154.4 days in Spain to 184.7 days in Türkiye, with an average of 169.9 days (Figure 1). During this period, the coldest temperatures were observed in the two continental GBs, Türkiye (mean temperature 9.37–11.22 °C) and Spain (10.71–11.77 °C), with an overall average of 12.690 °C across the 16 site-years (Figure 2A; Table S3). Conversely, Morocco (14.98–15.98 °C) and Greece (13.54–15.29 °C) experienced the mildest winters. Cumulative precipitation during Phase A was highest in Italy (610.2 mm in 2021) and Türkiye (420.4–477.4 mm), while Spain and Morocco experienced drier winter periods with precipitations around 139.0–272.7 mm in Spain and 127.3–295.3 mm in Morocco (Figure S5A). Median daily solar radiation during Phase A was highest in Morocco (~14.7 kW-hr/m^2^/day) and lowest in Italy (~9.2 kW-hr/m^2^/day) (Figure S5B).

Phase B—from Full Budburst, *FB*_50_ to Full Flowering, *FF*_50_: the mean duration of Phase B was shortest in Morocco (37.1 days) and longest in Italy (53.2 days), with a total average of 46.6 days (Figure 1). During this time window, mean temperatures ranged from 16.55–21.58 °C in Morocco to 12.10–15.99 °C in Spain, with an overall average of 15.88 °C (Figure 2B; Table S3). During this phase, maximum daily temperatures reached 24.41–30.22 °C in Morocco, while they remained lower in Italy (18.18–19.29 °C) and Greece (16.52–20.14 °C). In contrast, minimum daily temperatures during Phase B were lowest in Spain (ranging from 6.97 °C to 8.59 °C) and highest in Greece (ranging from 11.56 °C to 14.48 °C). Solar radiation in Phase B was consistently highest in Morocco and Türkiye and lowest in Spain and Italy (Figure S5B).

### 2.3. Characterization of Budburst and Flowering Dynamics

**Figure 3:**
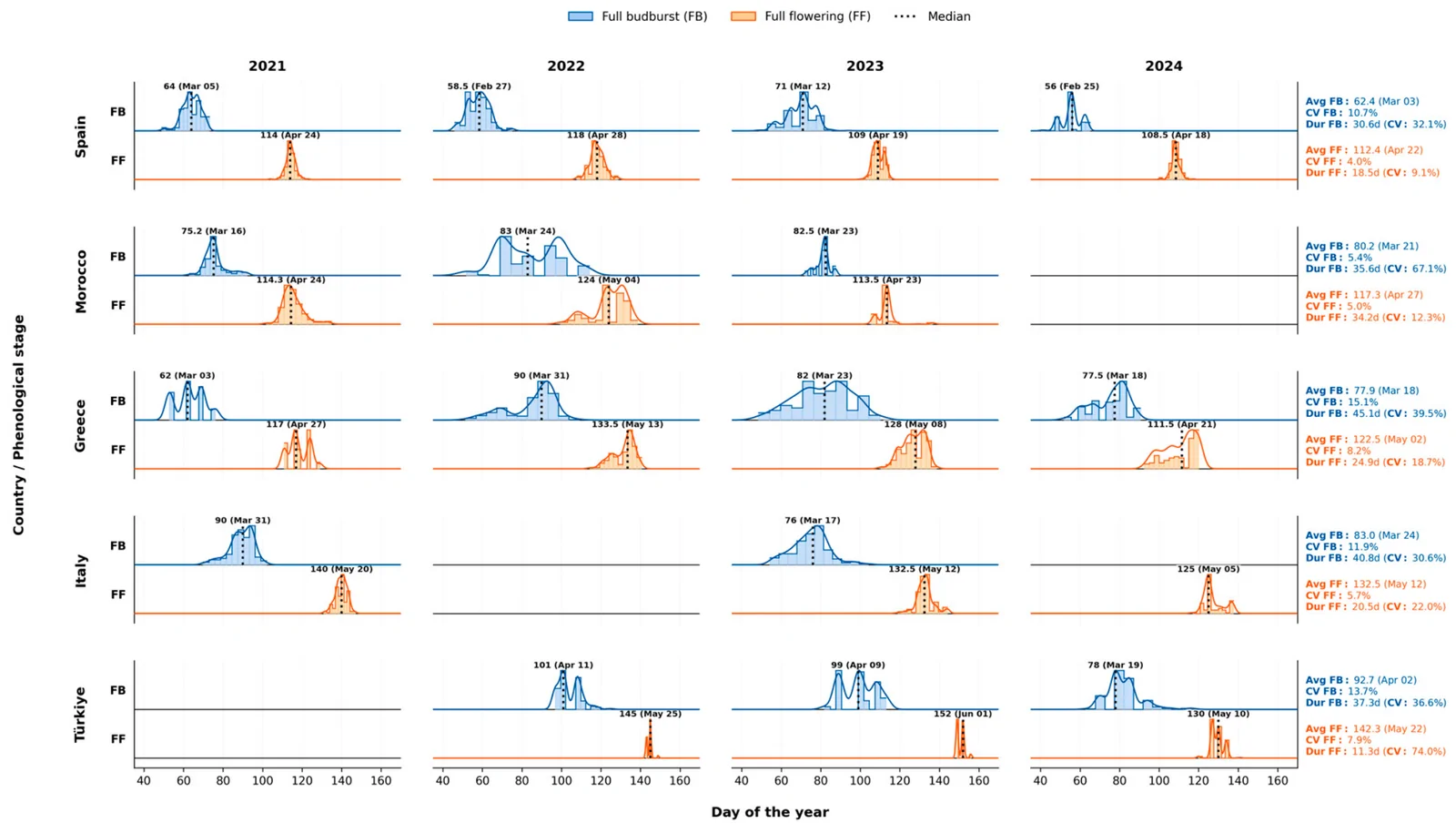
Collection-level distributions of Full Budburst (FB) and Full Flowering (FF) dates across Mediterranean germplasm-bank environments and years. Note: ridgeline plots show Full Budburst (blue) and Full Flowering (orange) dates. Dotted lines indicate medians; summaries show the average phenological stage, coefficient of variation (CV), and budburst or flowering duration (Dur) period. DOY, day of the year.

**Figure 4:**
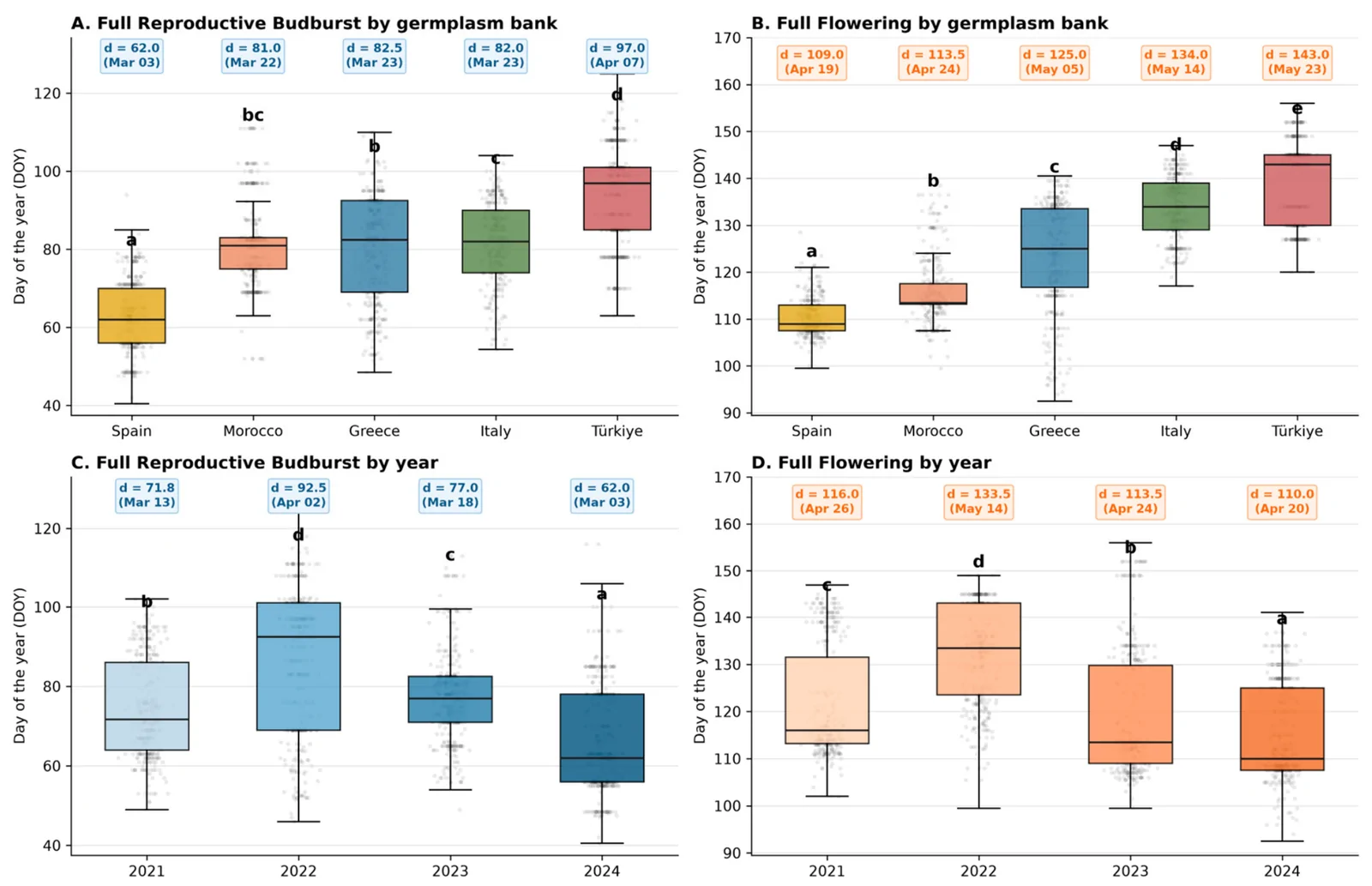
Environmental and interannual variation in Full Budburst (FB) and Full Flowering (FF) dates. Note: FB and FF dates are shown by country—germplasm-bank environment (**A**,**B**) and year (**C**,**D**). Groups sharing a letter did not differ in Tukey-adjusted pairwise comparisons from the linear mixed-effects model at *α* = 0.05. DOY, day of the year. The median DOY value is indicated in each box (d).

The timing of Full Budburst (*FB*_50_) and Full Flowering (*FF*_50_) varied significantly across locations and study years (Figure 3 and Figure 4 and Figure S6; Table S4).

Overall, we observed a West-to-East gradient in the timing of *FB*_50_ and *FF*_50_ (Figure 4; Tables S5 and S6). Both phenological phases differed significantly among sites (ANOVA; *p* < 0.0001). Spain and Türkiye represented the extremes, with Spain being the earliest and Türkiye the latest for both stages. Specifically, dates differed by 35 days for budburst (DOY 62.0 vs. DOY 97.0, respectively) and 34 days for flowering (DOY 109.0 vs. DOY 143.0) (Figure 4A,B).

Interannual variability was also pronounced across the dataset. The exceptionally warm 2024 stood out as the earliest for both stages across the five GBs (*FB*_50_ DOY 62.0 and *FF*_50_ DOY 110.0). In contrast, the cooler year of 2022 was consistently the latest, showing an average delay relative to 2024 of 30.5 days for budburst (*FB*_50_ DOY 92.5) and 23.5 days for flowering (*FF*_50_ DOY 133.5) (Tables S5 and S7; Figure 4).

**Figure 5:**
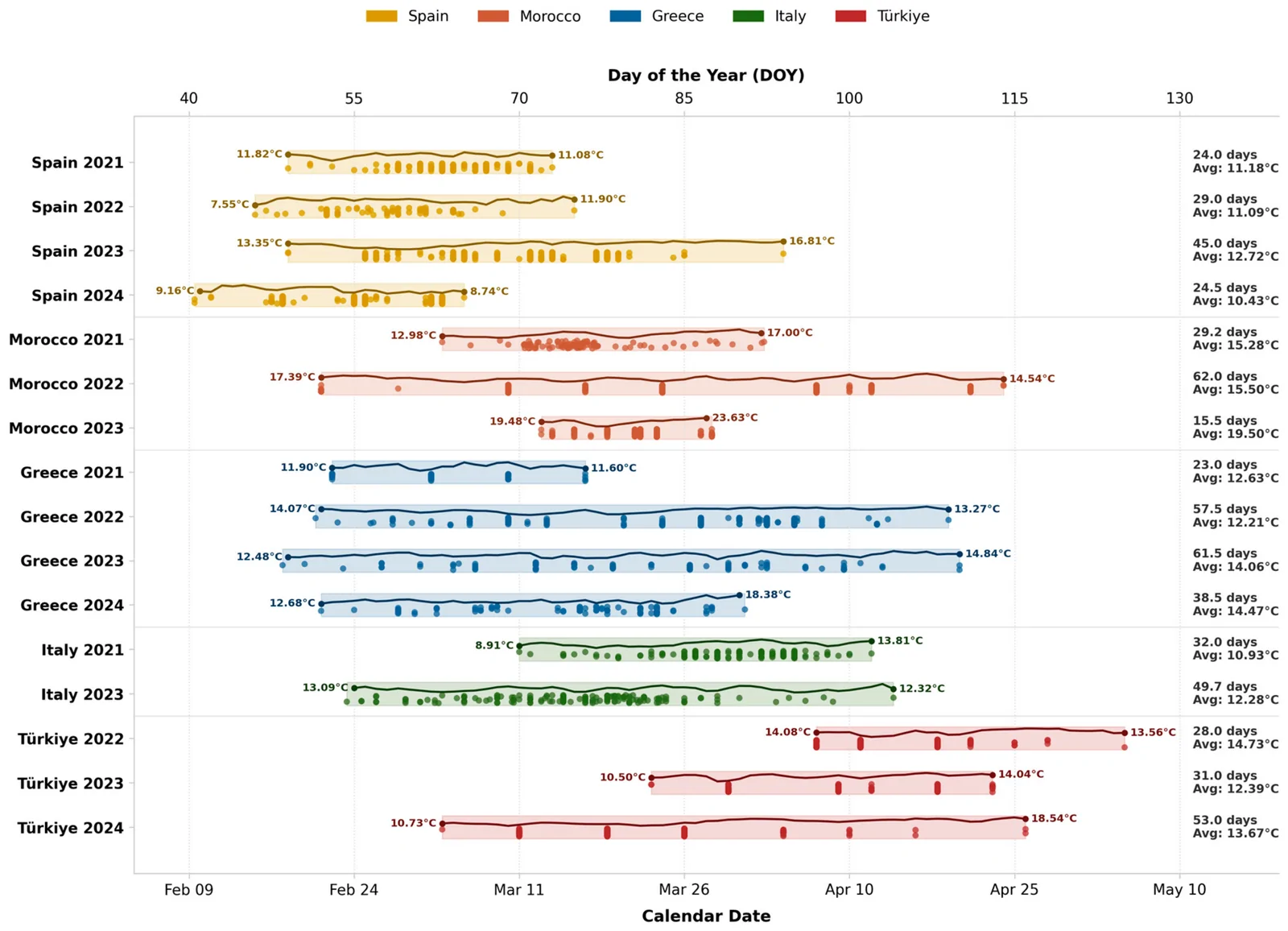
Collection-level budburst periods and daily temperature trajectories across Mediterranean germplasm-bank environments and years. Note: points represent individual-cultivar Full Budburst dates. Shaded bands span the budburst period (earliest to latest observation), and lines show mean daily temperature during that period.

**Figure 6:**
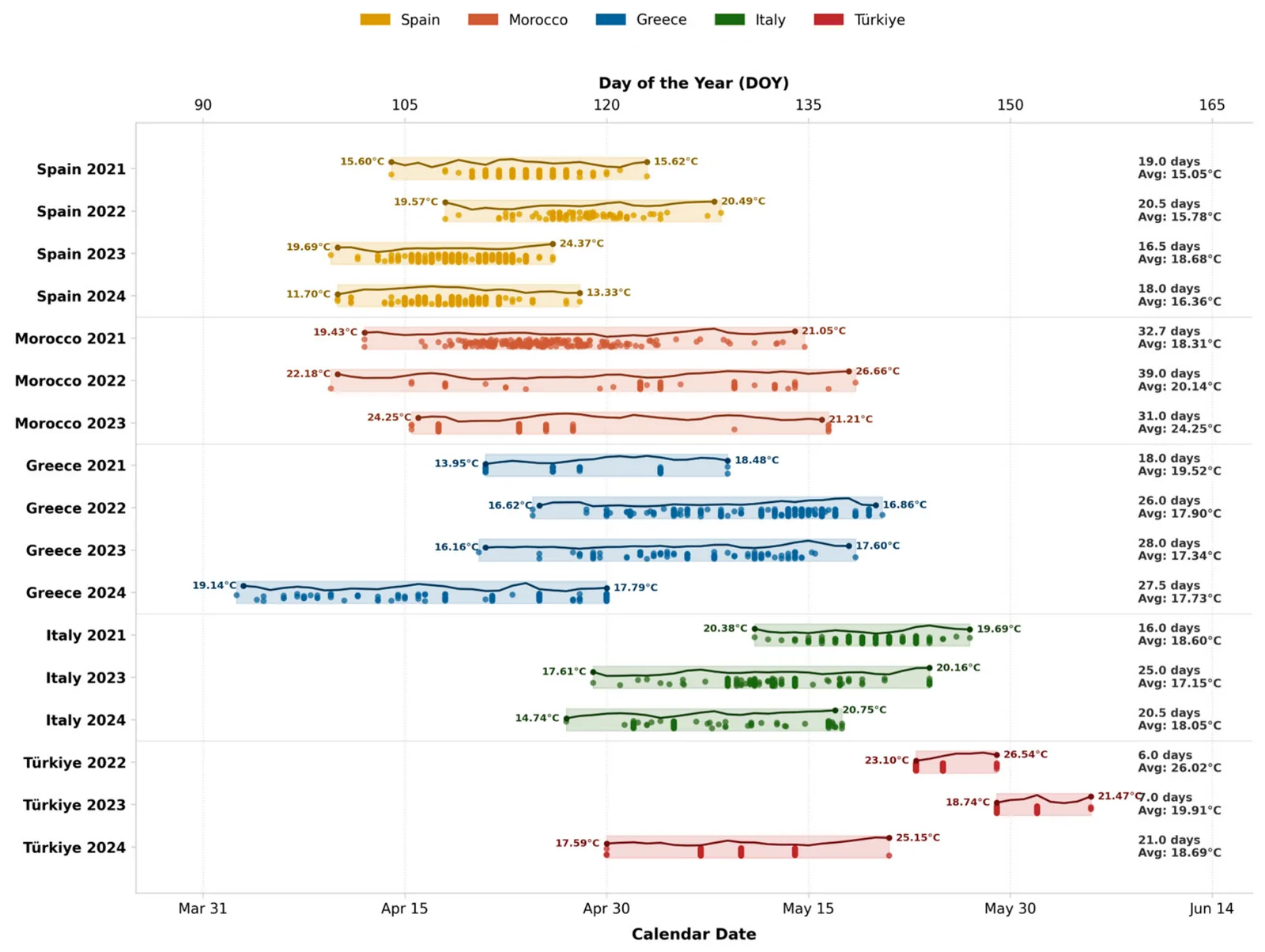
Collection-level flowering periods and daily temperature trajectories across Mediterranean germplasm-bank environments and years. Note: points represent individual-cultivar Full Flowering dates. Shaded bands span the flowering period (earliest to latest observation), and lines show mean daily temperature during that period.

Across the 16 site-years, the mean *FB*_50_ occurred on 19 March (DOY 77.86) and mean *FF*_50_ on 4 May (DOY 124.43; Table 1). The earliest median date for both stages, *FB*_50_ and *FF*_50_, was recorded in Spain in 2024 (*FB*_50_ DOY 56.0; *FF*_50_ DOY 108.5). Conversely, the latest *FB*_50_ occurred in Türkiye in 2022 (DOY 101.0), and the latest *FF*_50_ occurred in Türkiye in 2023 (DOY 152.0). Mean temperature during the observed FB period ranged from 10.4 °C in Spain in 2024 to 19.5 °C in Morocco in 2023. During the observed FF period, it ranged from 15.1 °C in Spain in 2021 to 26.0 °C in Türkiye in 2022 (Figure 5 and Figure 6; Table S4).

Beyond mean timing, the duration of these phenological phases also varied substantially. Across all site-years, the average budburst period was 37.7 days, while the flowering period averaged 21.9 days. Overall, the duration of reproductive budburst and flowering differed among environments and years, with both events lasting longer in environments or years characterized by milder and/or warmer climatic conditions (Table S4).

Across the 16 site-years, *ID_bb_* averaged 19.28 days, ranging from 13.75 days in Spain to 26.53 days in Greece. Meanwhile, *ID_ff_* averaged 10.24 days (Table 1), with the lowest values in Türkiye (4.00 days) and Spain (6.66 days), an intermediate value in Italy (7.83 days), and the highest values in Morocco (15.03 days) and in Greece (16.09 days). The maximum flowering dispersion occurred in Morocco in 2022 (*ID_ff_* = 25.80 days) and Greece in 2024 (22.50 days), whereas Türkiye recorded the minimum in 2022 (*ID_ff_* of 2.00 days).

The interval between *FB*_50_ and *FF*_50_ (*IM*) averaged 46.57 days across all observations, ranging from 31.0 days in Morocco (2023) to 59.5 days in Spain (2022).

The transition gap (*T_gap_*), which describes the temporal coexistence of budburst and flowering stages within a collection and year, was positive in Spain, Italy, and Türkiye, with country means of 26.12, 22.00, and 19.33 days. These positive values indicate a clear temporal separation between both phases with no overlap. *T_gap_* peaked at 35 days in Spain in 2024 and 36 days in Türkiye in 2023. Conversely, Morocco and Greece showed negative or near zero mean *T_gaps_* in specific years, peaking with a 14.5 day overlap in 2022 in Morocco (Table 1).

### 2.4. Chilling and Thermal Indices

**Figure 7:**
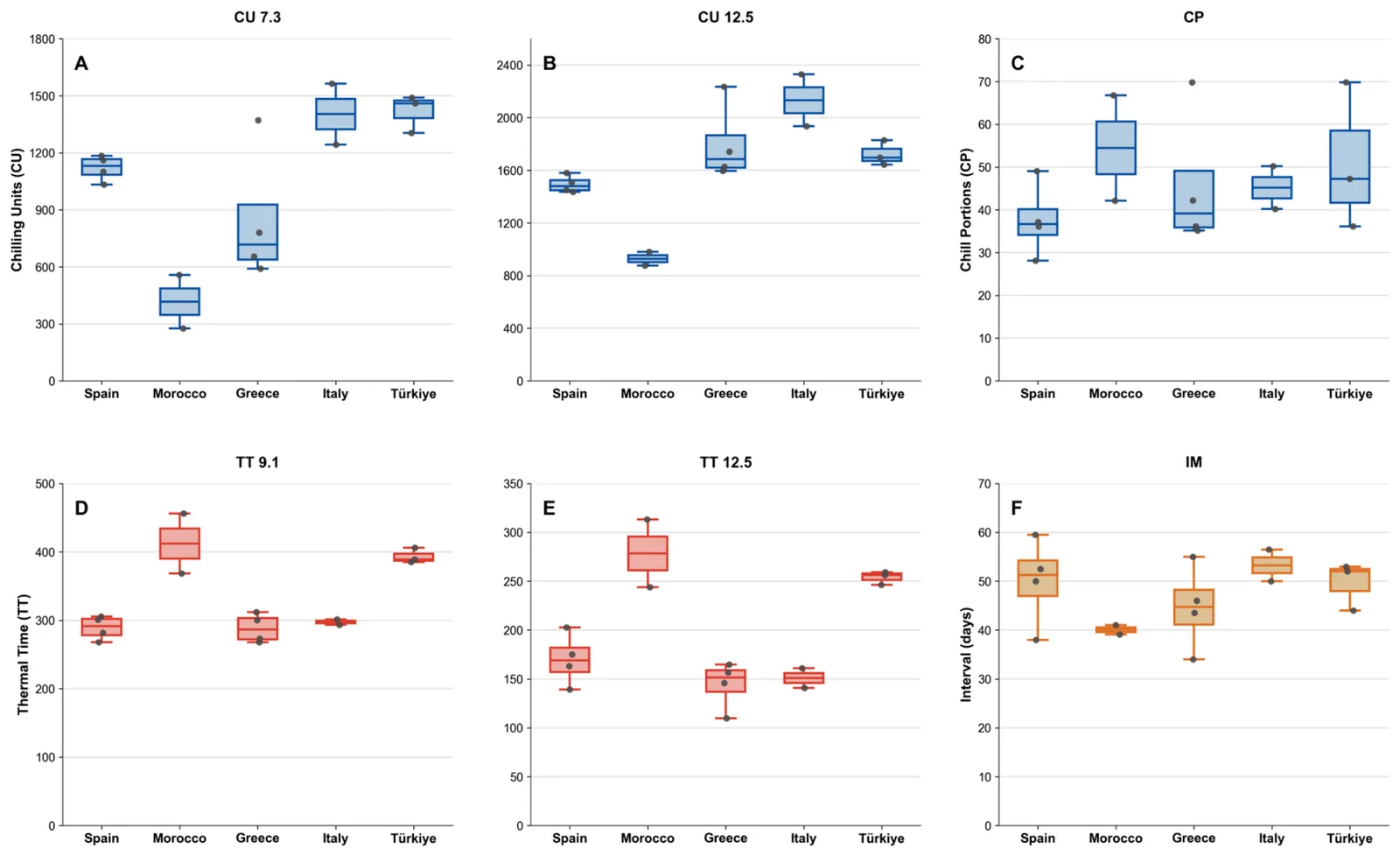
Chilling accumulation (Phase A), thermal time accumulation (Phase B), and the median budburst-to-flowering interval (IM) across Mediterranean germplasm-bank environments. Note: panels show (**A**) *CU*_7.3_, (**B**) *CU*_12.5_, (**C**) CP, (**D**) *TT*_9.1_, (**E**) *TT*_12.5_, and (**F**) the median FB-to-FF interval (*IM*). Points represent site-years; boxes show the median and interquartile range. *CU*_7.3_ and *CU*_12.5_, chilling units accumulated from 1 October to FB_50_ using optima of 7.3 and 12.5 °C; CP, chill portions; and TT_9.1_ and TT_12.5_, thermal time accumulated from FB_50_ to FF_50_ using base temperatures of 9.1 and 12.5 °C.

The accumulation of chilling and thermal time showed significant variation across the Mediterranean network (Table 1; Figure 7).

Across the 16 site-years, mean chilling accumulation was 45.50 CP, 1011.77 CU_7.3_, and 1586.93 CU_12.5_ (Table 1; Figure 7A–C). The relative ranking of the germplasm-bank environments varied among chilling indices. Türkiye and Italy showed the highest mean CU_7.3_ values (1418.34 and 1404.08 units, respectively), whereas Morocco showed the lowest CU_7.3_ accumulation (416.08 units). The highest mean CP was in Türkiye (51.06), closely followed by Morocco (50.25). Italy recorded the highest CU_12.5_ accumulation (2133.39 units), while Greece also accumulated more CU12.5 than Türkiye (1800.24 vs. 1723.34 units), despite its lower CU_7.3_ value. These contrasting rankings illustrate that the chilling indices describe different components of the winter thermal regime. Relative variability among site-years was lower for CU_12.5_ (CV = 0.260) and CP (CV = 0.282) than for CU_7.3_ (CV = 0.406).

The thermal forcing accumulated during Phase B showed a different geographical pattern (Table 1; Figure 7D,E). Across the network, mean accumulation was 326.00 TT_9.1_ and 194.94 TT_12.5_. Türkiye recorded the highest mean forcing value (393.39), followed by Morocco (376.97), whereas Morocco recorded the highest mean TT_12.5_ value (265.75), followed by Türkiye (254.05). Lower TT_12.5_ values were observed in Spain, Italy, and Greece (170.08, 150.96, and 144.35, respectively).

### 2.5. Factors Associated with Budburst and Flowering Dynamics

We examined two fixed factors associated with the timing of olive reproductive FB and FF across the studied sites: germplasm-bank environment (country), interannual variation (year), and their interaction (country × year) (Table S8; Figure 4). According to the two-way factorial ANOVA, both main effects and their interaction were highly significant for both phenological stages (*p* < 0.0001). The effect magnitude was quantified using the partial eta-squared (ηp^2^). Country showed the largest partial effect size for both stages (FB: ηp^2^ = 0.581; FF: ηp^2^ = 0.821). Year also showed substantial partial effect sizes (FB: ηp^2^ = 0.272; FF: ηp^2^ = 0.492), as did the country × year interaction (FB: ηp^2^ = 0.211; FF: ηp^2^ = 0.180). The significant interaction indicates that interannual differences in phenological timing varied among germplasm-bank environments. A complementary linear mixed-effects analysis supported the broad effects of the germplasm-bank environment (country) and year identified via ANOVA (Table S9), as both effects remained significant. After accounting for the country and year, consistent differences among cultivars represented approximately 40.0% of the remaining variation in Full Budburst timing and 26.3% in Full Flowering timing (intraclass correlation coefficients, ICC = 0.400 and 0.263, respectively). Overall, the mixed-model results reinforced the main environmental and interannual effects.

**Figure 8:**
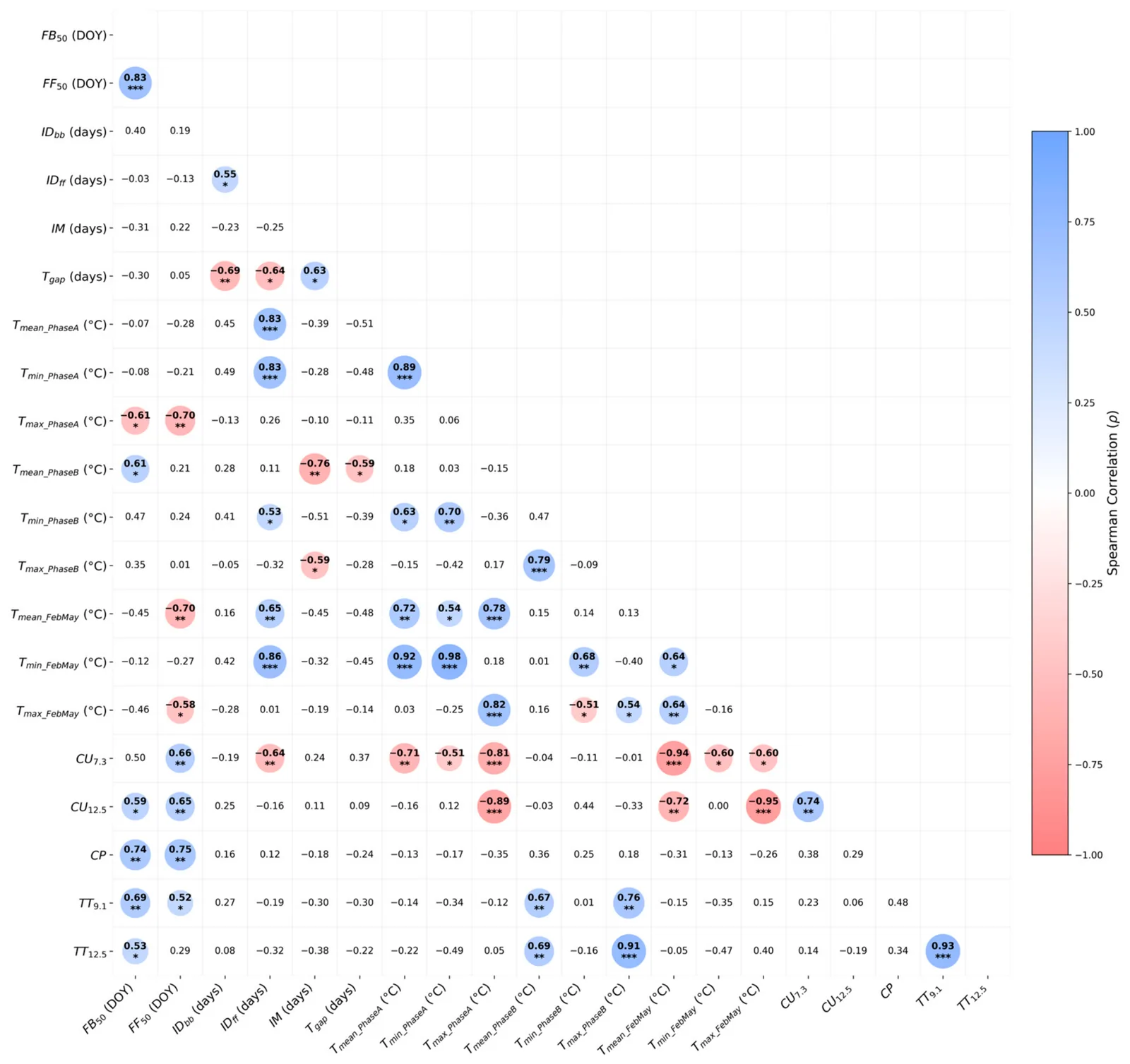
Spearman rank correlations among collection-level phenological and thermal variables. Note: significant correlations are shown by circles (blue, positive; red, negative); *, **, and *** indicate *p* < 0.05, *p* < 0.01, and *p* < 0.001, respectively. Abbreviations: *ID_bb_* and *ID_ff_*, budburst and flowering dispersion; *IM*, median FB-to-FF interval; *T_gap_*, transition gap; CP, chill portions; CU, chilling units; TT, thermal time; and FebMay, 1 February–31 May. *CU*_7.3_ and *CU*_12.5_, chilling units accumulated from 1 October to FB_50_ using optima of 7.3 and 12.5 °C; CP, chill portions; and TT_9.1_ and TT_12.5_, thermal time accumulated from FB_50_ to FF_50_ using base temperatures of 9.1 and 12.5 °C.

To further characterize the relationship between budburst and flowering, as well as their associations with bioclimatic variables and temperature regimes across the Mediterranean Basin, phenological indices were correlated with bioclimatic parameters and temperature regimes across 16 site-years at the collection level (Figure 8).

This analysis showed a strong positive correlation between the onset of budburst and flowering across the Mediterranean GBs. At the population level, the median budburst (*FB*_50_) and flowering date (*FF*_50_) were highly positively correlated (*ρ* = 0.83, *p* < 0.001). Furthermore, this dependency extended to the budburst and flowering dispersion (*ID_bb_* and *ID_ff_*, respectively), which were also significantly positively correlated (*ρ* = 0.55, *p* < 0.05). These results describe close collection-level correlation between the timing and dispersion of the two reproductive stages.

Regarding the specific environmental variables, both phenological stages showed associations with winter chilling accumulation. However, the Dynamic Model (CP) and the mild-winter model (*CU*_12.5_) were the only chilling accumulation indices significantly correlated with both phenological phases. Specifically, CP exhibited the strongest positive correlations with the median budburst date (*FB*_50_, *ρ* = 0.74, *p* < 0.01) and flowering date (*FF*_50_, *ρ* = 0.75, *p* < 0.01), followed by *CU*_12.5_ (*FB*_50_, *ρ* = 0.59, *p* < 0.01; *FF*_50_, *ρ* = 0.65, *p* < 0.01). In contrast, the De Melo-Abreu chilling units (*CU*_7.3_) did not show significant correlations with the onset of budburst. Nevertheless, it showed a significant correlation with the flowering timing (*FF*_50_, *ρ* = 0.66, *p* < 0.01).

Finally, response patterns under warmer winter conditions indicated that the variable *CU*_7.3_ was particularly sensitive to elevated temperatures. It showed strong negative correlations with temperatures within the winter windows, such as the pre-flowering fixed window *T_mean_*__Feb-May_ (*ρ* = −0.94, *p* < 0.001) and *T_max_*__PhaseA_ (*ρ* = −0.81, *p* < 0.001). Consequently, the minimum chilling accumulation occurred in warm-winter years, such as Greece in 2024 (*CU*_7.3_ = 590.71) and Morocco in 2022 (*CU*_7.3_ = 276.42). This contrasted sharply with the high chilling accumulated in cold-winter environments like Italy in 2021 (*CU*_7.3_ = 1564.37), Türkiye in 2022 (*CU*_7.3_ = 1490.26), and Spain in 2021 (*CU*_7.3_ = 1184.49).

This variation in chilling accumulation was also associated with flowering synchrony. The flowering dispersion (*ID_ff_*) was significantly negatively correlated with *CU*_7.3_ (*ρ* = −0.64, *p* < 0.01) and positively correlated with mean *T_mean_*__phaseA_ (*ρ* = 0.83, *p* < 0.001). Thus, across the observed site-years, greater CU_7.3_ accumulation was associated with narrower flowering dispersion, whereas warmer Phase A conditions were associated with broader dispersion. For instance, in Spain in 2024 (*T_mean_*__PhaseA_ = 11.77 °C, *CU*_7.3_ = 1032.68) and Türkiye in 2022 (*T_mean_*__PhaseA_ = 9.37 °C, *CU*_7.3_ = 1490.26), flowering took place in just a few days (*ID_ff_* of 5 and 2 days, respectively). However, under elevated winter temperatures, the subsequent flowering period tended to be more extended. For instance, in Greece in 2024, where winter warming was severe (*T_mean_*__PhaseA_ = 15.29 °C, *CU*_7.3_ = 590.71), the flowering dispersion was particularly high (*ID_ff_* = 22.50 days). On the other hand, no significant correlation was detected between ID_ff_ and chill portions (CP; *ρ* = 0.12, *p* = 0.677).

The median interval between *FB*_50_ and *FF*_50_ (IM) was negatively correlated with both mean and maximum temperatures during Phase B (*T_mean_*__PhaseB_, *ρ* = −0.76, *p* < 0.01; *T_max_*__PhaseB_, *ρ* = −0.59, *p* < 0.05). Therefore, warmer Phase B conditions were associated with a shorter interval between budburst and flowering.

Similarly, the transition gap (*T_gap_*) between the end of budburst and the beginning of flowering was negatively correlated with mean Phase B temperature (*T_mean_*__PhaseB_, *ρ* = −0.59, *p* < 0.05) and negatively correlated with flowering dispersion (*ID_ff_*, *ρ* = −0.64, *p* < 0.01). A negative *T_gap_*, indicates overlap between budburst and flowering within the collection (e.g., Morocco in 2022 with *T_gap_* = −14.5 days). Across the observed site-years, warmer Phase B conditions were therefore associated with greater phase overlap and wider dispersion. On the other hand, the thermal time (*TT*_12.5_) was positively correlated with the median budburst date (*FB*_50_, *ρ* = 0.53); however, because TT_12.5_ was accumulated between FB_50_ and FF_50_, this descriptive association is partly influenced by the phenology-dependent window and should not be interpreted independently.

## 3. Discussion

### 3.1. Regional Climates and Phenological Timing Across the Mediterranean

Evaluating olive cultivars across five international germplasm banks in Spain, Morocco, Greece, Italy, and Türkiye confirmed a broad macroclimatic gradient across the Mediterranean Basin. Bioclimatic classification combining WBCS and Bioclim variables [29,30] structured the network into contrasting continental regimes with marked daily thermal oscillations and warm maritime microclimates [31].

Evaluating olive phenology in this macroecological context overcomes the limitations of single-location or geographically narrow studies [11,16]. As warming and erratic winter thermal regimes increasingly disrupt chilling accumulation in traditional growing areas [4,32], germplasm-bank networks provide a more standardized framework to assess phenotypic variation and adaptive potential in perennial crops, including olives [17]. Adopting a population approach across these collections moves beyond isolated cultivar evaluations. By treating these repositories as representative populations of the crop’s genetic diversity, our analysis captures collective reproductive dynamics. This provides an empirical baseline to assess regional adaptation patterns and climatic vulnerability across the Mediterranean basin.

Across our germplasm-bank network, phenological monitoring revealed a clear progression consistent with the sequential action of winter chilling and spring heat. In continental regimes, phenological progression was associated with marked daily thermal ranges and seasonal temperature shifts. Earlier spring warming in Spain coincided with the earliest median budburst (*FB*_50_) and Full Flowering (*FF*_50_) in the network. Conversely, lower late-winter and early-spring temperatures in continental Türkiye delayed budburst by nearly a month, shifting both phenophases to the latest network positions.

In maritime-influenced regimes, mild winters produced distinct responses. Moderate seasonal temperatures in Italy enabled steady phenological progress, whereas mild winters in Greece coincided with later initial budburst. In thermally extreme, arid Morocco, elevated winter and spring temperatures, combined with high daily thermal oscillations, were associated with irregular budburst timing. Patterns in Morocco and Greece parallel observations in subtropical Tenerife and Argentina, where warm winters were associated with asynchronous flowering [4,9,33].

Overall, budburst exhibited greater spatial and temporal variation than Full Flowering, with mean budburst dispersion (*IDbb* = 19.28 days) being approximately twice the mean flowering dispersion (*IDff* = 10.24 days). This flowering population-level synchronization may represent an adaptive feature that enhances cross-pollination efficiency in olive, a wind-pollinated and self-incompatible species [28]. In contrast, warm-winter conditions were associated with asynchronous phenological overlap, resulting in negative *Tgap* values in Morocco and values approaching zero in Greece, where late budburst coexisted with early flowering within the same collection, as also reported in subtropical environments [4,33].

### 3.2. Evaluation of Chilling Accumulation Models Across Macroclimatic Regimes

Chilling models differ fundamentally in how they integrate temperature: fixed-threshold models [34] assume linear accumulation near 7.3 °C, whereas Dynamic Models (CP) account for a two-step process in which intermediate products can be reversed by high temperatures [35]. These physiological distinctions explain performance contrasts between maritime-buffered sites such as Greece and the thermally extreme, arid conditions of Morocco. Across our network, accumulated chilling (CU_7.3_) averaged 1011.77 CU (CV = 0.406), falling within the range reported by Belaj et al. [11] in Spain (635–1188 CU_7.3_).

The interpretation of these accumulations depends directly on the definition of each interval. In this study, chilling accumulation was calculated for Phase A from 1 October to the observed *FB*_50_, whereas TT was accumulated during Phase B, from *FB*_50_ to *FF*_50_. This phenological delimitation differs from approaches that use fixed calendar periods or a common calendar cut-off for the onset of forcing [34,36]. Anchoring each thermal window to observed phenological dates captures the actual thermal accumulation experienced by the collections, although it inherently couples the window duration with developmental timing. Because these thermal windows depend on local phenological observations and vary across different environments, independent forcing experiments under controlled conditions remain necessary to isolate and determine the true intrinsic chilling requirements of each cultivar [37]. Consequently, our CU and TT values should not be compared directly with estimates obtained from fixed windows or interpreted as interchangeable physiological requirements [38].

This macroecological evaluation highlights the limits of fixed chilling thresholds [4,9]. In subtropical Tenerife, minimum winter temperatures rarely drop below standard thresholds (e.g., 7.3), leading classical models to estimate near-zero chilling despite consistent flowering [4]. In our study, we observed a comparable pattern in Greece, where buffered maritime conditions yielded mean CU_7.3_ accumulation of 849.25 units. However, because temperatures hovered around 15 °C, shifting the threshold to 12.5 °C (CU_12.5_) increased accumulation in Greece to 1800.24 CU. This suggests that the 7.3 °C model may not fully capture the thermal window governing floral induction in olives, which ranges approximately from 9 to 16 °C [6].

In Morocco, warm winter days with pronounced daily thermal oscillations were associated with a low mean CU_7.3_ accumulation of 416.08 chilling units, consistent with chilling negation at high temperatures [34]. Under the Dynamic Model, Morocco accumulated an average of 50.25 CP, close to the highest network maximum. Abou-Saaid and Khadari [39] noted that standard model parameters may underestimate olive chilling requirements, establishing baseline values of approximately 58 CP for medium-chilling and 12 CP for low-chilling cultivars. Relative to the network mean (45.50 CP; CV = 0.282), several studied locations would fall below this benchmark for medium-chilling cultivars, emphasizing the need for local calibration. Overall, models incorporating broader thermal inductive windows (CU12.5) and dynamic formulations (CP) described complementary aspects of thermal accumulation across Mediterranean macroclimates.

Interpreting the macroclimatic patterns observed across the 16 site-years requires careful consideration. Because chilling accumulation was calculated up to the observed FB50, the interval duration was inherently linked to local phenological timing. Furthermore, because the analysis reflects the collective response of each collection, these results represent descriptive exploratory trends rather than intrinsic requirements for individual cultivars or transferable causal mechanisms.

### 3.3. Factors Influencing Phenological Synchrony and Flowering Dynamics

One of the strongest correlations occurred between budburst timing (FB_50_) and Full Flowering timing (FF_50_) (*ρ* = 0.83, *p* < 0.001). Likewise, flowering dispersion (ID*_ff_*) was significantly and positively correlated with budburst dispersion (ID*_bb_*) and with the fixed-window pre-flowering temperature (T*_mean_*__*Feb*_*May*._). Across the observed site-years, higher chilling accumulation was associated with late budburst and greater flowering synchrony among cultivars. Conversely, low chilling accumulation was associated with higher budburst and flowering dispersion [4,33,40].

Biologically, winter chilling accumulation is required for floral induction in olive buds, after which heat accumulation contributes to ecodormancy release and subsequent development towards anthesis [6,7,8,41]. Across the observed site-years, greater chilling accumulation sometimes coincided with later budburst. This pattern is biologically plausible because colder and/or longer winters permit greater chilling accumulation but may also delay the subsequent accumulation of the thermal time required for budburst; it should not be interpreted as evidence that chilling itself delays budburst. Progression toward Full Flowering was also associated with Phase B temperature and thermal accumulation [4,16,25,42].

The transition gap (T*_gap_*) was negatively correlated with the mean Phase B temperature. Continental locations with sufficient chilling maintained positive T*_gap_* values, reflecting temporal separation between budburst and flowering phases. In contrast, warm-winter environments exhibited negative or near-zero T*_gap_* values, with late budburst coexisting with early flowering within the collection [4,33,40].

From a practical perspective, reproductive budburst provides a useful temporal reference for evaluating collection-level phenological progression and potential flowering asynchrony. Linking budburst and flowering dispersion through the transition gap offers a quantitative framework for comparing flowering synchrony in warm-winter conditions [4,33]. As warming winters and frequent warm spells increasingly interrupt cold accumulation [32,43], selecting genotypes with high positive T*_gap_* values may represent an effective adaptation strategy in future studies. At the collection level, ANOVA results indicated that while climatic conditions represented the primary effect for both stages, budburst exhibited lower environmental sensitivity than flowering. Nevertheless, this distinction should be evaluated in-depth using shared cultivars with contrasting phenological responses across different collections. Such comparisons would help determine whether wide dispersion reflects stable genetic differences or inadequate local adaptation and exposure to fluctuating winter and spring temperatures [25,26,33].

## 4. Materials and Methods

### 4.1. Germplasm Collections and Environmental Data

#### 4.1.1. Germplasm Bank Location and Bioclimatic Characterization

Phenology was monitored from 2021 to 2024 in 670 cultivars maintained in five international germplasm banks (GBs) across the Mediterranean Basin (Figure S1): ELGO-DEMETER in Chania, Greece (79 cultivars; 35.49° N, 24.03° E); CREA in Mirto Crosia, Italy (148 cultivars; 39.62° N, 16.77° E); WOGB-UCO in Córdoba, Spain (192 cultivars; 37.93° N, 4.72° W); INRA in Marrakech, Morocco (171 cultivars; 31.82° N, 7.43° W); and ORI in İzmir, Türkiye (80 cultivars; 38.46° N, 27.37° E). At least two trees were evaluated per cultivar in each germplasm bank. Because each country was represented by one bank, the country factor also represents the corresponding germplasm-bank environment.

The number of cultivars with complete phenological observations varied among site-years (Table S4), reflecting differences in the collection size and data availability. Consequently, collection-level medians and dispersion estimates derived from smaller samples may have lower precision, and comparisons among site-years were interpreted with this unequal sampling structure in mind.

The environmental characterization of the five GBs was carried out in two steps. First, their macroclimatic profiles were classified using the Worldwide Bioclimatic Classification System [29] and the regional definitions adapted for the Euro-Mediterranean region according to [31]. Daily maximum, minimum, and mean temperature and precipitation were obtained from NASA POWER for 1981–2025. The following WBCS indicators were calculated according to [29]: the Continentality Index (*I_c_*), Annual Ombrothermic Index (*I_o_*), and Compensated Thermicity Index (*I_tc_*) (Table S1).

Second, a multivariate statistical approach was used to validate and complement the qualitative WBCS classifications. To do so, a Principal Component Analysis (PCA) was applied to the 19 standard bioclimatic variables (BIO1–BIO19) calculated for each site-year according to [30] (Table S2). Daily temperature and precipitation were aggregated into monthly values, and the bioclimatic variables were calculated with the biovars function in the R package dismo (version 1.3-14) [44], following WorldClim definitions [30]. Variables were centered and scaled before PCA with the prcomp function in R. Site-year scores, collection centroids, 95% confidence ellipses, and environmental loading vectors were projected onto the first two principal components (Figure S2).

#### 4.1.2. Fixed and Dynamic Climatic Windows

A fixed pre-flowering window from 1 February to 31 May was used to compare mean, minimum, and maximum temperatures among germplasm-bank environments and years. The starting date of 1 February was selected because previous olive phenological models have used it as a fixed reference for the onset of post-winter thermal accumulation, approximately representing the transition from the main chilling period to pre-flowering development under Mediterranean conditions [12,34]. This interval was used as a standardized calendar reference rather than as an assumed universal date of dormancy release.

Two phenology-dependent climatic windows were also defined for each site-year:

Phase A: from 1 October of year *N* − 1 to the median Full Budburst date (*FB*_50_) in year *N*. This window covered the period used to calculate chilling accumulation before budburst [34].

Phase B: from *FB*_50_ to the median Full Flowering date (*FF*_50_) in year *N*. This window covered post-budburst development and heat accumulation before flowering.

Because *FB*_50_ and *FF*_50_ differed among GBs and years, the end points and duration of both phases varied among site-years.

The phase-specific windows were defined to provide biologically aligned, retrospective descriptions of the thermal conditions experienced before each observed phenological stage. However, because *FB*_50_ and *FF*_50_ determine the end points and durations of these windows, the resulting phase-specific temperature and thermal-accumulation variables are mathematically coupled with the phenological dates.

### 4.2. Phenological Monitoring and Derived Metrics

Budburst and flowering were monitored across the five GBs with a shared protocol based on the BBCH scale adapted for olive [45,46] and used within the Gen4olive H2020 project [45,46,47]. Observations covered the entire tree canopy and recorded the most delayed, most common, and most advanced stages as the vertices of a phenological triangle [48]. Reproductive budburst (BBCH stages 51–54, with stage 53 representing Full Budburst) was assessed weekly, whereas flowering (BBCH stages 60–69, with stage 65 representing Full Flowering) was assessed every 2–3 days, following the Gen4olive protocol [47].

For each cultivar, Full Budburst and Full Flowering were calculated as the average DOYs of the beginning and end of Full Budburst and Flowering, respectively, with BBCH 65 defining full bloom. For each site-year, the median dates of FB and FF across all observed cultivars were denoted as *FB*_50_ and *FF*_50_, respectively. Medians were used to reduce the influence of extreme observations.

The phenological dispersion of budburst and flowering at the collection level was quantified as the interdecile interval of FB (*ID_bb_*) and FF (*ID_ff_*), to minimize the influence of outliers:

*ID_bb_* = *P*_90,*FB*_ − *P*_10,*FB*_

*ID_ff_* = *P*_90,*FF*_ − *P*_10,*FF*_

where *P*_10_ and *P*_90_ are the 10th and 90th percentiles, respectively, of the FB or FF dates within each site-year. Smaller values indicate greater concentration of observations around the median.

The budburst and flowering periods were defined as the number of days between Full Budburst/Flowering of the earliest and latest cultivars for each site-year.

The interval between *FF*_50_ and *FB*_50_ (*IM*, days) was calculated as follows:

*IM* = *FF*_50_ − *FB*_50_

The transition gap (*T_gap_*, days) described separation or overlap between the two stages:

*T*_*gap*_ = *FF*_*min*_ − *FB*_*max*_

where *FF_min_* is the earliest FF date and *FB_max_* is the latest FB date within a site-year. Positive *T_gap_* values indicate temporal separation between stages, whereas negative values indicate overlap within the collection.

### 4.3. Chilling and Forcing Quantification

Daily maximum and minimum temperatures from NASA POWER were interpolated using trigonometric functions to hourly values. The daily minimum was assigned to sunrise and the maximum to two hours after solar noon; sunrise and solar-noon times were calculated for each location and day with the R package suncalc (version 0.5.1).

Three chilling formulations were calculated from 1 October of year *N −* 1 to *FB*_50_ in year *N*:

Dynamic Model: chilling portions were calculated with the two-step Dynamic Model of [49]. In the first step, intermediate cold temperatures promote the formation of an unstable precursor (*S*). High temperatures reverse this process, degrading the precursor. In the second step, when the precursor concentration reaches a critical threshold, it converts irreversibly into a chilling portion (CP). The temperature dependence enters through the reaction rates, which are assumed to follow specific Arrhenius laws. To compute the amount of CP, the required system of kinetic ordinary differential equations was built and solved at hourly timesteps using the original parameters and the algorithm of [49].

Chilling units with a 7.3 °C optimum (*CU*_7.3_): using the model of De Melo-Abreu et al. [34], hourly temperatures were used to compute a response function that assigns greater weight to temperatures around 7.3 °C, negatively weighting temperatures above 20.7 °C (chill negation). The values of the function are added for each hour to aggregate the total number of chilling units, discarding negative totals.

Chilling units with a 12.5 °C optimum (*CU*_12.5_): the same response function was used in this case but with the maximum displaced to 12.5 °C to represent chilling under milder winter temperatures, using the intermediate inductive temperature range of 9 to 16 °C proposed for olives [6].

The two chilling unit formulations were calculated as follows:

$$CU_{x}=\sum_{h=1}^{24D}f_{x}(T_{h}),\;\;x\in \{7.3,12.5\}$$

where *D* is the number of days from 1 October to *FB*_50_, *T_h_* is hourly air temperature, and *f_x_* is the temperature-response function centered at *x* °C.

Post-budburst heat accumulation (forcing) was computed from *FB*_50_ to *FF*_50_ with base temperatures (*T_b_*) of 9.1 °C and 12.5 °C:

$$TT=\frac{1}{24}\sum_{h=FB_{50}}^{FF_{50}}max(0,T_{h}-T_{b}),\;\;T_{b}\in \{9.1,12.5\}$$

The resulting variables were denoted as *TT*_9.1_ and *TT*_12.5_.

### 4.4. Statistical Analyses

Statistical analyses were performed in R (version 4.4.1). The R packages used for linear mixed-effects models, Type III analyses of variance, and post-hoc comparisons were lme4 (version 1.1-37), lmerTest (version 3.1-3), car (version 3.1-3), emmeans (version 1.10.0), and multcomp (version 1.4-26). Graphics produced in R used ggplot2 (version 3.5.2) and cowplot (version 1.1.3).

#### 4.4.1. Variation Among Germplasm Banks and Years

Two-way factorial analysis of variance (ANOVA) was used to evaluate the effects of the germplasm-bank environment (factor country), year, and their interaction on the cultivar-level FB and FF dates. Residual normality and homogeneity of variance were assessed with Shapiro–Wilk and Levene tests. Neither assumption was met for the untransformed day of the year (DOY). Following square-root transformation, the ANOVA residuals satisfied the assumptions of normality and homogeneity of variance, with no significant departures detected by either diagnostic test (*p* > 0.05); therefore, √DOY was used in the factorial ANOVA. The independence assumption was considered separately in relation to the sampling structure.

As a complementary analysis, linear mixed-effects models were fitted to the same observations, with the germplasm-bank environment and year as fixed effects and cultivar identity as a random grouping factor. Post-hoc pairwise comparisons of the estimated marginal means from the mixed-effects models, with a Tukey adjustment for multiple comparisons, were used to generate the compact-letter groupings shown in Figure 4.

Because the multi-site experiment contained missing data (e.g., no budburst records for Italy in 2022 or Morocco in 2024), Type III sums of squares were obtained via nested model comparisons and used to accommodate the unbalanced factorial structure. The partial eta-squared ($\eta_{p}^{2}$) was reported as an effect-size measure.

#### 4.4.2. Associations Between Phenology and Climatic Conditions

Spearman rank correlations were calculated across the 16 available site-years to describe associations among *FB*_50_, *FF*_50_, *ID_bb_*, *ID_ff_*, *IM*, *T_gap_*, chilling and forcing indices, phase-specific temperatures, and fixed-window temperatures. Two-tailed *p*-values were evaluated at *α* = 0.05. Because the same five germplasm-bank environments were represented across multiple years, the site-years were not assumed to be fully independent. Furthermore, CP, CU_7.,_ and CU_12.5_ were accumulated from 1 October to FB_50_, whereas TT_9.1_ and TT_12.5_ were accumulated from FB_50_ to FF_50_. Correlations involving these phenology-dependent indices were therefore interpreted as descriptive comparisons of the thermal conditions experienced during the observed phases, rather than as independent predictive or causal relationships.

## 5. Conclusions

Coordinated monitoring across five Mediterranean olive germplasm banks showed that reproductive budburst and flowering varied substantially among bank environments and years. The relative temporal dispersion observed at reproductive budburst was largely maintained through Full Flowering, while the post-budburst temperature was associated with the rate of progression between stages. Likewise, greater budburst dispersion (ID*_bb_*) was associated with greater flowering dispersion (ID*_ff_*), indicating a close collection-level relationship between the synchrony of both reproductive stages.

Evaluating the median timing of budburst and flowering together with the budburst dispersion (ID*_bb_*), flowering dispersion (ID*_ff_*), and transition gap (T*_ga_*_p_) revealed changes in the phenological coordination and phase overlap that the median the flowering date alone cannot detect. These metrics may assist comparative assessments of phenological variation and climate adaptation in olive genetic resources. Applying them to future breeding programs will require an evaluation of shared cultivars and breeding progenies across multi-environment trials to separate genetic traits from local climate influences.

Our findings show that regional climatic differences across the Mediterranean basin were associated not only with phenological timing, but also with the temporal dispersion and synchrony of reproductive phenology in olive collections. Furthermore, ANOVA confirmed that the environment played an important role in variation in both phenophases; budburst was considerably less sensitive to environmental conditions than flowering. Moreover, wider phenological dispersion occurred particularly under warmer winter conditions, where reduced chilling accumulation was associated with less synchronous development. Nevertheless, because each country was represented by a single germplasm bank, the environmental effect cannot be attributed exclusively to climate, and the component associated with cultivars should not be interpreted as a strict estimate of genetic variance.

## Figures and Tables

**Table 1:** Collection-level phenology, dispersion, developmental intervals, and thermal accumulation across 16 site–years.

Site–Year	FB_50_ (DOY)	ID_bb_ (Days)	FF_50_ (DOY)	ID_ff_ (Days)	IM (Days)	T_gap_ (Days)	CU_7.3_	CU_12.5_	CP	TT_9.1_	TT_12.5_
Greece 2021	62.00	16.70	117.00	13.00	55.00	35.00	654.18	1627.30	35.13	267.87	109.75
Greece 2022	90.00	29.50	133.50	13.88	43.50	5.50	1371.92	2235.10	69.81	312.18	164.86
Greece 2023	82.00	36.10	128.00	15.00	46.00	0.50	780.21	1742.94	42.19	300.06	145.92
Greece 2024	77.50	23.80	111.50	22.50	34.00	2.00	590.71	1595.61	36.15	273.46	156.88
Italy 2021	90.00	14.00	140.00	7.00	50.00	29.00	1564.37	2330.35	50.19	301.27	161.09
Italy 2023	76.00	22.67	132.50	8.65	56.50	15.00	1243.80	1936.44	40.19	293.58	140.83
Morocco 2021	75.21	13.26	114.34	11.29	39.14	9.75	556.83	980.56	42.15	368.42	243.88
Morocco 2022	83.00	33.00	124.00	25.80	41.00	−14.50	276.42	875.96	66.80	456.10	313.13
Morocco 2023	82.50	7.50	113.50	8.00	31.00	18.00	415.00	920.00	41.80	422.38	341.80
Spain 2021	64.00	10.00	114.00	6.00	50.00	31.00	1184.49	1580.74	36.13	301.19	175.04
Spain 2022	58.50	11.50	118.00	9.15	59.50	33.00	1161.25	1436.92	49.05	267.83	139.34
Spain 2023	71.00	19.00	109.00	6.50	38.00	5.50	1101.38	1507.55	37.17	305.69	202.86
Spain 2024	56.00	14.50	108.50	5.00	52.50	35.00	1032.68	1451.35	28.13	281.73	163.08
Türkiye 2022	101.00	14.00	145.00	2.00	44.00	18.00	1490.26	1697.90	69.81	385.11	259.36
Türkiye 2023	99.00	19.00	152.00	3.00	53.00	36.00	1459.88	1828.45	47.22	406.21	256.41
Türkiye 2024	78.00	24.00	130.00	7.00	52.00	4.00	1304.87	1643.66	36.15	388.85	246.38
Mean Greece	77.88	26.53	122.50	16.09	44.62	10.75	849.25	1800.24	45.82	288.39	144.35
Mean Italy	83.00	18.33	136.25	7.83	53.25	22.00	1404.08	2133.39	45.19	297.42	150.96
Mean Morocco	80.24	17.92	117.28	15.03	37.05	4.42	416.08	925.51	50.25	376.97	265.75
Mean Spain	62.38	13.75	112.38	6.66	50.00	26.12	1119.95	1494.14	37.62	289.11	170.08
Mean Türkiye	92.67	19.00	142.33	4.00	49.67	19.33	1418.34	1723.34	51.06	393.39	254.05
Global Mean	77.86	19.28	124.43	10.24	46.57	16.42	1011.77	1586.93	45.50	326.00	194.94

Note: median dates of Full Budburst (FB_50_) and Full Flowering (FF_50_); ID_bb_ and ID_ff_, intervals between the 10th and 90th percentiles of FB and FF; IM = FF_50_ − FB_50_; Tgap = FFmin-FBmax; DOY, day of the year; CU_7.3_ and CU_12.5_, chilling units accumulated from 1 October to FB_50_ using optima of 7.3 and 12.5 °C; CP, chill portions; and TT_9.1_ and TT_12.5_, thermal time accumulated from FB_50_ to FF_50_ using base temperatures of 9.1 and 12.5 °C.

## Data Availability

The processed data supporting the findings of this study will be provided with the article and its Supplementary Materials. Additional data may be made available by the corresponding author upon justified request and subject to the data-governance requirements of the participating institutions.

## Supplementary Materials

The following supporting information can be downloaded at: https://www.mdpi.com/article/10.3390/plants15192980/s1. Figure S1: Geographic locations of the five Mediterranean olive germplasm banks. Note: Stars indicate WOGB-UCO (Spain), INRA (Morocco), ELGO-DEMETER (Greece), CREA (Italy), and ORI (Türkiye). Figure S2: Principal component analysis of long-term bioclimatic variation across the five germplasm-bank environments. Note: The analysis used 19 bioclimatic variables calculated from NASA POWER data for 1981–2025. Points represent years, ellipses 95% confidence regions, diamonds collection centroids, and arrows variable loadings. Figure S3: Monthly temperature and precipitation profiles at the five germplasm banks during 2021–2024. Note: Black lines show median monthly temperature, shaded bands the minimum–maximum temperature range, and blue bars cumulative monthly precipitation. Data were obtained from NASA POWER. Figure S4: Mean temperatures and temperature ranges during the fixed February–May window. Note: Diamonds show mean temperature and vertical bars the mean daily minimum–maximum range from 1 February to 31 May. Shaded columns show multi-year or network means. Figure S5: Phase-specific precipitation and solar radiation across Mediterranean germplasm-bank environments and years. Note: (A) Cumulative precipitation and (B) median daily solar radiation during Phase A and Phase B. Phase A extends from 1 October to FB_50_, and Phase B from FB_50_ to FF_50_. Figure S6: Germplasm-bank distributions of Full Budburst and Full Flowering dates within each study year. Note: Boxplots show Full Budburst (upper row) and Full Flowering (lower row) dates by environment and year. DOY, day of the year. Table S1: Long-term bioclimatic characteristics of the five germplasm-bank environments (1981–2025). Table S2: Loadings of the 19 bioclimatic variables on the first four principal components. Table S3: Phase-specific and fixed-window temperatures across the 16 site–years. Table S4: Descriptive phenological statistics by germplasm-bank environment and year. Table S5: Phenological timing and Tukey HSD groupings by germplasm-bank environment and year. Table S6: Tukey HSD pairwise comparisons among germplasm-bank environments, pooled across years. Table S7: Tukey HSD pairwise comparisons among years, pooled across germplasm-bank environments. Table S8: Effects of germplasm-bank environment, year, and their interaction on Full Budburst and Full Flowering dates. Table S9: Complementary linear mixed-effects analysis of Full Budburst and Full Flowering timing across olive germplasm-bank environments and years.

## Abbreviations

The following abbreviations are used in this manuscript:

General abbreviations	
ANOVA	Analysis of variance
BBCH	Biologische Bundesanstalt, Bundessortenamt und Chemische Industrie phenological scale
BIO1–BIO19	Standard bioclimatic variables 1–19
CRAN	Comprehensive R Archive Network
CREA	Council for Agricultural Research and Economics
CREA-OFA	Council for Agricultural Research and Economics—Research Centre for Olive, Fruit and Citrus Crops
CV	Coefficient of variation
DOY	Day of the year
ELGO-DEMETER	Hellenic Agricultural Organization–DIMITRA
GB	Germplasm bank
H2020	Horizon 2020
HSD	Honestly significant difference
INRA	Institut National de la Recherche Agronomique
IOC	International Olive Council
NASA POWER	National Aeronautics and Space Administration Prediction Of Worldwide Energy Resources
ORI	Olive Research Institute
PCA	Principal Component Analysis
PC1; PC2	First and second principal components, respectively
SS	Sum of squares
UCO	Universidad de Córdoba
WBCS	Worldwide Bioclimatic Classification System
WOGB-UCO	World Olive Germplasm Bank of the Universidad de Córdoba
Phenological, climatic, and statistical variables	
CP	Chill portions
*CU*_7.3_	Chilling units calculated using a 7.3 °C optimum
*CU*_12.5_	Chilling units calculated using a 12.5 °C optimum
FB	Full Budburst
*FB*_50_	Median Full Budburst date
*FB_max_*	Latest Full Budburst date within a site-year
FF	Full Flowering
*FF*_50_	Median Full Flowering date
*FF_min_*	Earliest Full Flowering date within a site-year
*I_c_*	Continentality Index
*I_o_*	Annual Ombrothermic Index
*I_tc_*	Compensated Thermicity Index
*ID_bb_*	Interdecile interval of Full Budburst dates
*ID_ff_*	Interdecile interval of Full Flowering dates
IM	Median interval between the median Full Budburst and median Full Flowering dates
*P*_10,*FB*;*P*_90,*FB*_	10th and 90th percentiles of Full Budburst dates, respectively
*P*_10,*FF*;*P*_90,*FF*_	10th and 90th percentiles of Full Flowering dates, respectively
*P_ann_*	Annual precipitation
*T_ann_*	Annual mean temperature
*T_gap_*	Transition gap between the latest Full Budburst and earliest Full Flowering observations
*T_mean_*_,*Feb–May*_	Mean temperature during the fixed 1 February–31 May window
*T_min_*_,*Feb–May*_	Minimum temperature during the fixed 1 February–31 May window
*T_max_*_,*Feb–May*_	Maximum temperature during the fixed 1 February–31 May window
*T_mean_*_,*Phase A*_	Mean temperature during Phase A
*T_max_*_,*Phase A*_	Maximum temperature during Phase A
*T_mean_*_,*Phase B*_	Mean temperature during Phase B
*T_max_*_,*Phase B*_	Maximum temperature during Phase B
*TT*_9.1_	Thermal time accumulated above 9.1 °C between the median Full Budburst and median Full Flowering dates
*TT*_12.5_	Thermal time accumulated above 12.5 °C between the median Full Budburst and median Full Flowering dates
