# Supplementary material for: Budburst and Flowering Dynamics of Cultivated Olive Across the Mediterranean Basin
Source: Plants (Basel). 2026 Sep 29;15(19):2980. doi: 10.3390/plants15192980 (PMC13667179; doi:10.3390/plants15192980)
Supplement: Supplementary file 1 [file plants-15-02980-s001.zip › Supplementary figures.pdf]

Article

Budburst and Flowering Dynamics of Cultivated Olive Across the Mediterranean Basin

Rüştü Efe Değer[1,†], Hristofor Miho [1,†], Esteban Meca [2], Diego Cabello Pozo [1], Georgios Koubouris [3], Ioanna Manolikaki [3], Sara Oulbi [4], Omar Abou-Saaid [4], Enzo Perri [5], Samanta Zelasco [5], Hülya Kaya [6], Songül Acar [6] and Concepción Muñoz Díez [1,*]

Supplementary Figures

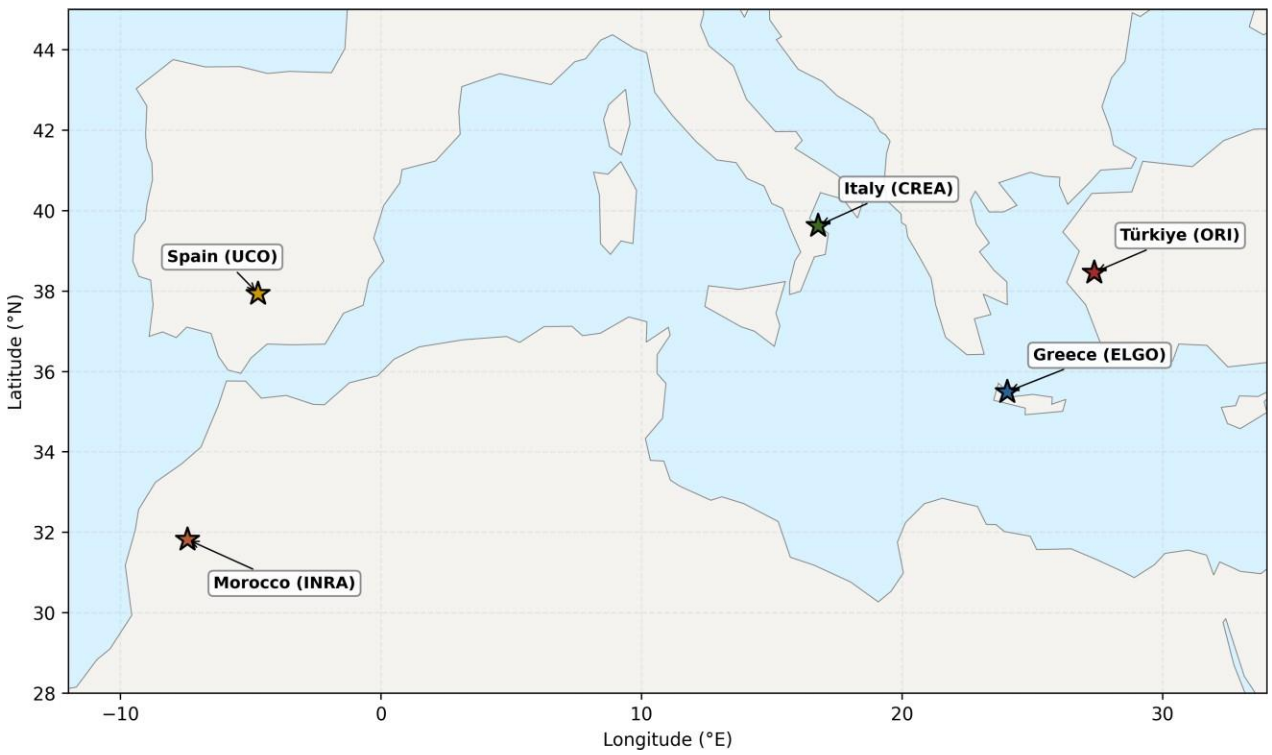

Figure S1. Geographic locations of the five Mediterranean olive germplasm banks.

Note: Stars indicate WOGB-UCO (Spain), INRA (Morocco), ELGO-DEMETER (Greece), CREA (Italy) and ORI (Türkiye).

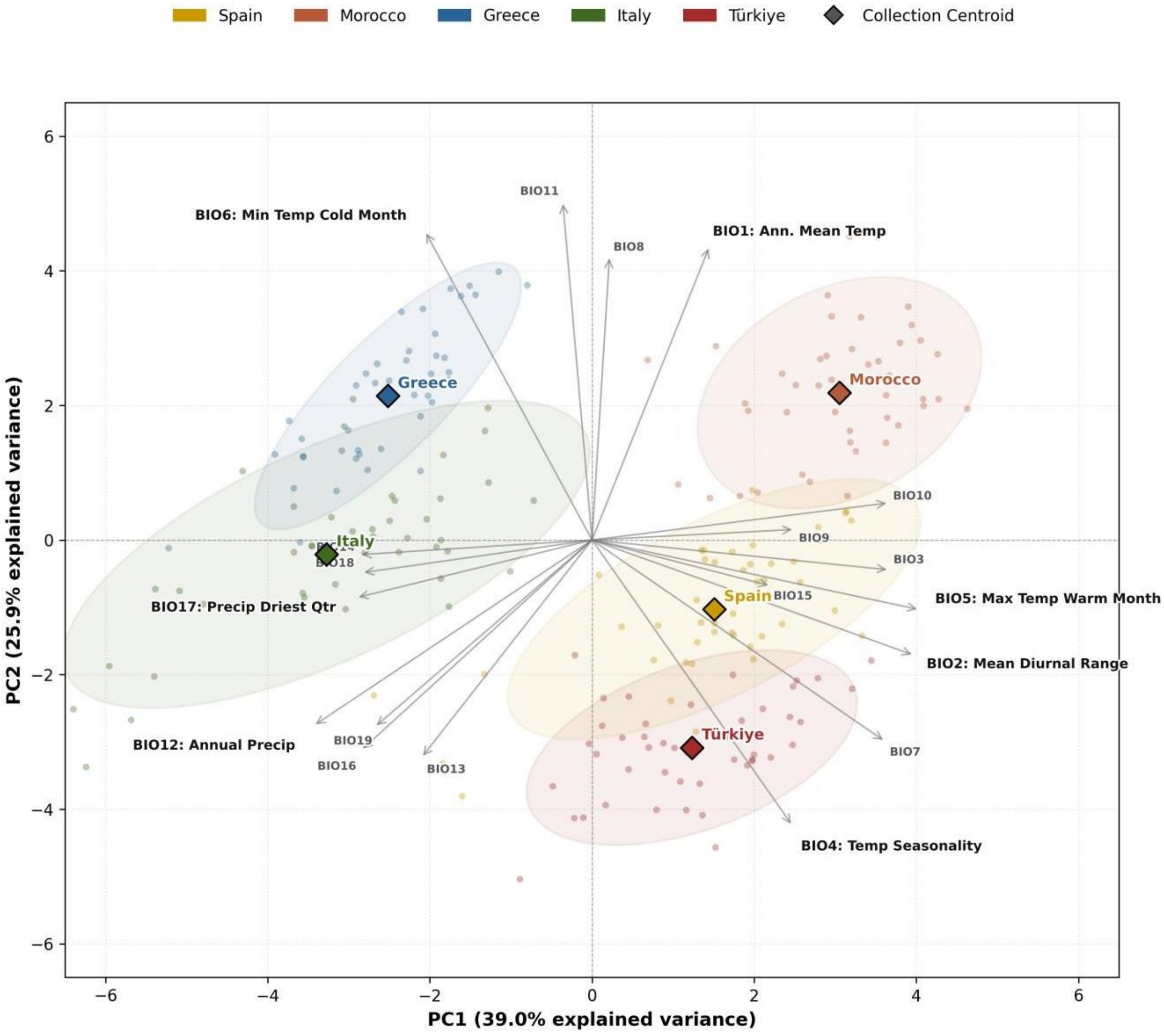

Figure S2. Principal component analysis of long-term bioclimatic variation across the five germplasm-bank environments.

Note: The analysis used 19 bioclimatic variables calculated from NASA POWER data for 1981–2025. Points represent years, ellipses 95% confidence regions, diamonds collection centroids and arrows variable loadings.

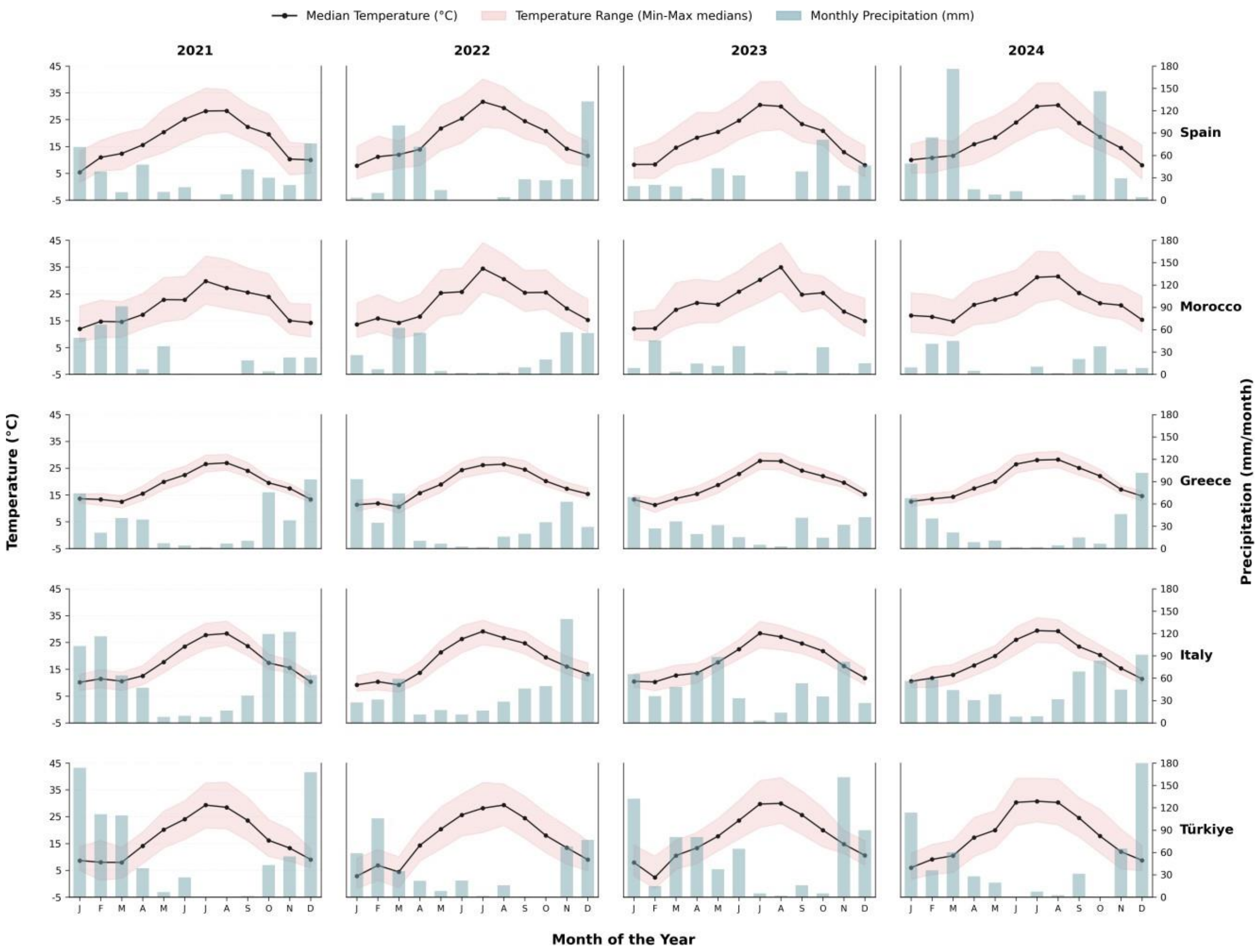

Figure S3. Monthly temperature and precipitation profiles at the five germplasm banks during 2021–2024.

Note: Black lines show median monthly temperature, shaded bands the minimum–maximum temperature range, and blue bars cumulative monthly precipitation. Data were obtained from NASA POWER.

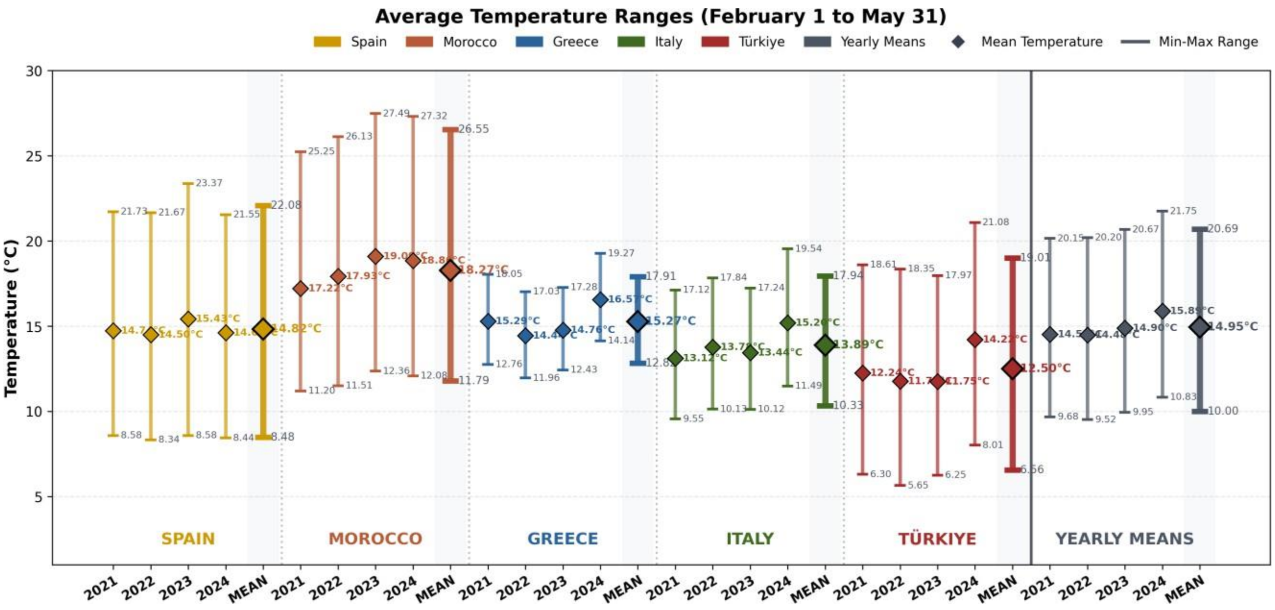

Figure S4. Mean temperatures and temperature ranges during the fixed February–May window.

Note: Diamonds show mean temperature and vertical bars the mean daily minimum–maximum range from 1 February to 31 May. Shaded columns show multi-year or network means.

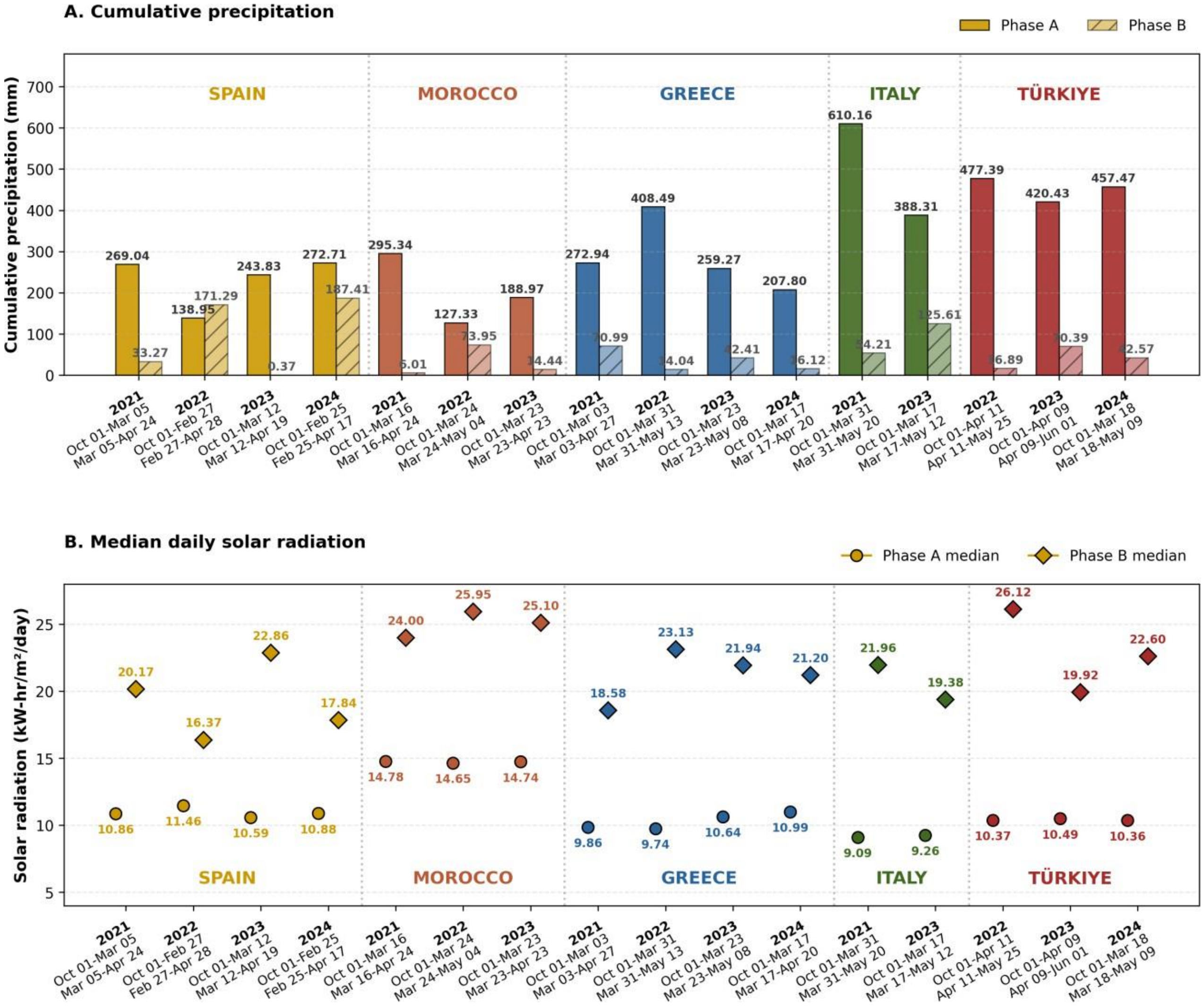

Figure S5. Phase-specific precipitation and solar radiation across Mediterranean germplasm-bank environments and years.

Note: (A) Cumulative precipitation and (B) median daily solar radiation during Phase A and Phase B. Phase A extends from 1 October to FB_{50}, and Phase B from FB_{50} to FF_{50}.

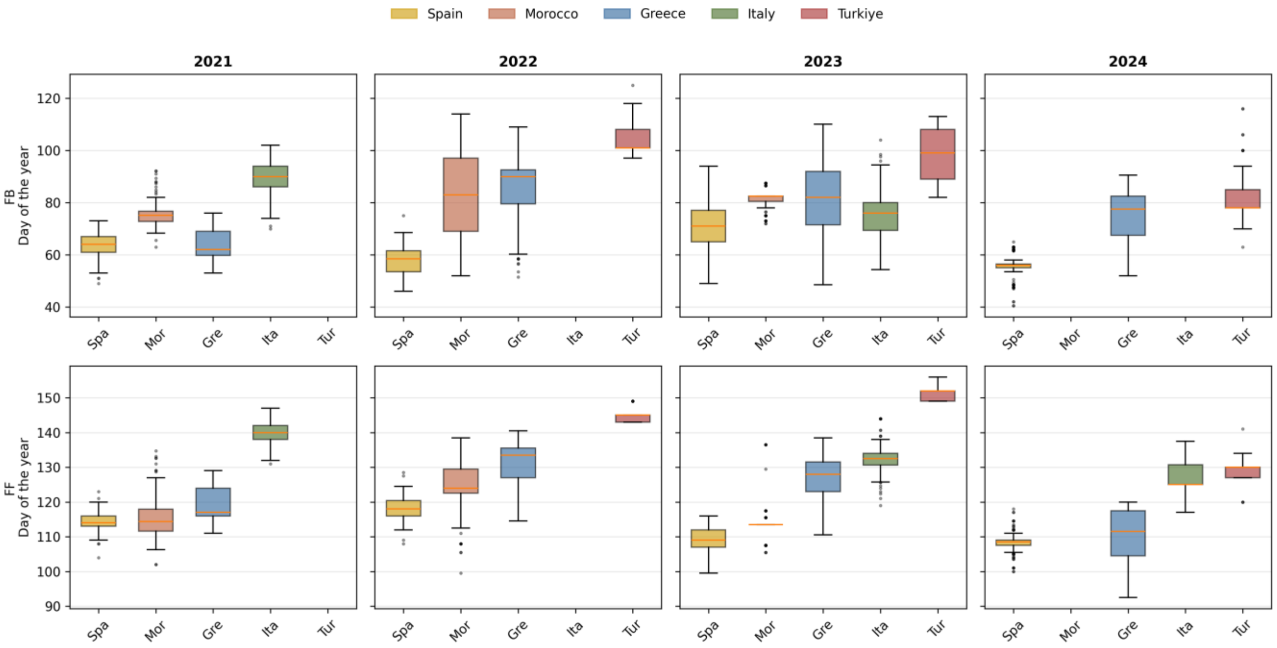

Figure S6. Germplasm-bank distributions of Full Budburst and Full Flowering dates within each study year.

Note: Boxplots show Full Budburst (upper row) and Full Flowering (lower row) dates by environment and year. DOY, Day of the Year.
