# Supplementary material for: Budburst and Flowering Dynamics of Cultivated Olive Across the Mediterranean Basin
Source: Plants (Basel). 2026 Sep 29;15(19):2980. doi: 10.3390/plants15192980 (PMC13667179; doi:10.3390/plants15192980)
Supplement: Supplementary file 1 [file plants-15-02980-s001.zip › Supplementary Tables.pdf]

Article

Budburst and Flowering Dynamics of Cultivated Olive Across the Mediterranean Basin

Rüştü Efe Değer[1,†], Hristofor Miho [1,†], Esteban Meca [2], Diego Cabello Pozo [1], Georgios Koubouris [3],

Ioanna Manolikaki [3], Sara Oulbi [4], Omar Abou-Saaid [4], Enzo Perri [5], Samanta Zelasco [5], Hülya Kaya [6],

Songül Acar [6] and Concepción Muñoz Díez [1,*]

Table S1. Long-term bioclimatic characteristics of the five germplasm-bank environments (1981–2025).

GB	Country	Coordinates	T_{ann} (°C)	P_{ann} (mm)	I_c	I_o	I_{tc}	WBCS classification (thermotype; ombrotype)
INRA	Morocco	31.82°N, 7.43°W	20.19	308.2	19.42	1.28	461.4	Med. Xeric Oceanic (Inframediterranean; Semi-arid)
WOGB-UCO	Spain	37.93°N, 4.72°W	16.77	474.3	21.52	2.37	349.9	Med. Pluviseasonal Continental (Mesomediterranean; Dry)
ELGO-DEMETER	Greece	35.49°N, 24.03°E	18.10	546.6	15.26	2.53	397.2	Med. Pluviseasonal Oceanic (Thermomediterranean; Dry)
CREA	Italy	39.62°N, 16.77°E	17.11	770.7	18.39	3.76	352.4	Med. Pluviseasonal Oceanic (Thermomediterranean; Sub-humid)
ORI	Türkiye	38.46°N, 27.37°E	16.16	595.9	24.05	3.08	329.2	Med. Pluviseasonal Continental (Mesomediterranean; Dry)

Note. WBCS, Worldwide Bioclimatic Classification System; GB, germplasm bank; T_{ann}, annual mean temperature; P_{ann}, annual precipitation; I_c, Continentality Index; I_o, Annual Ombrothermic Index; and I_{tc}, Compensated Thermicity Index.

Table S2. Loadings of the 19 bioclimatic variables on the first four principal components.

Code	Bioclimatic variable	PC1	PC2	PC3	PC4
BIO1	Annual Mean Temperature (°C)	0.1176	0.3542	0.0145	0.3545
BIO2	Mean Diurnal Range (°C)	0.3227	-0.1391	0.1472	0.1503
BIO3	Isothermality (BIO2/BIO7 × 100)	0.2974	-0.0360	0.1515	0.2271
BIO4	Temperature Seasonality (SD × 100)	0.2008	-0.3449	0.1112	-0.0754
BIO5	Max Temperature of Warmest Month (°C)	0.3279	-0.0841	0.1509	0.2324
BIO6	Min Temperature of Coldest Month (°C)	-0.1672	0.3727	-0.1098	0.1709
BIO7	Temperature Annual Range (BIO5-BIO6, °C)	0.2936	-0.2432	0.1518	0.0608
BIO8	Mean Temperature of Wettest Quarter (°C)	0.0175	0.3421	0.1386	0.1119
BIO9	Mean Temperature of Driest Quarter (°C)	0.2019	0.0126	-0.1742	0.1335
BIO10	Mean Temperature of Warmest Quarter (°C)	0.2969	0.0447	0.1280	0.2988
BIO11	Mean Temperature of Coldest Quarter (°C)	-0.0292	0.4082	-0.0470	0.2924
BIO12	Annual Precipitation (mm)	-0.2788	-0.2241	-0.0100	0.2208
BIO13	Precipitation of Wettest Month (mm)	-0.1706	-0.2624	-0.2063	0.3818
BIO14	Precipitation of Driest Month (mm)	-0.2324	-0.0177	0.4102	0.1286
BIO15	Precipitation Seasonality (CV)	0.1780	-0.0551	-0.3544	0.2597
BIO16	Precipitation of Wettest Quarter (mm)	-0.2312	-0.2537	-0.1916	0.2998
BIO17	Precipitation of Driest Quarter (mm)	-0.2354	-0.0695	0.4486	0.1708
BIO18	Precipitation of Warmest Quarter (mm)	-0.2298	-0.0394	0.4412	0.1536
BIO19	Precipitation of Coldest Quarter (mm)	-0.2174	-0.2255	-0.2182	0.2851

Note: BIO, standard bioclimatic variable; PC, principal component. Absolute loadings ≥0.25 identify variables with comparatively large contributions to a component.

Table S3. Phase-specific and fixed-window temperatures across the 16 site–years.

Site–year	Tmean,PhaseA (°C)	Tmin,PhaseA (°C)	Tmax,PhaseA (°C)	Tmean,PhaseB (°C)	Tmin,PhaseB (°C)	Tmax,PhaseB (°C)	Tmean,FebMay (°C)	Tmin,FebMay (°C)	Tmax,FebMay (°C)
Greece 2021	14.76	13.39	16.77	13.95	11.56	16.52	15.29	12.76	18.05
Greece 2022	13.54	11.79	15.25	16.23	13.41	19.29	14.44	11.96	17.03
Greece 2023	14.75	12.96	16.98	15.62	12.88	18.30	14.76	12.43	17.28
Greece 2024	15.29	13.75	17.41	17.20	14.48	20.14	16.57	14.14	19.27
Italy 2021	12.07	9.61	15.68	15.39	11.44	19.29	13.12	9.55	17.12
Italy 2023	12.86	10.07	16.55	14.05	10.23	18.18	13.44	10.12	17.24
Morocco 2021	14.98	9.78	22.46	17.21	12.10	25.39	17.22	11.20	25.25
Morocco 2022	15.25	9.94	23.07	16.55	9.99	24.41	17.93	11.51	26.13
Morocco 2023	15.98	10.57	24.01	21.58	13.67	30.22	19.09	12.36	27.49
Spain 2021	11.44	7.19	17.52	14.60	8.57	21.67	14.74	8.58	21.73
Spain 2022	10.71	5.54	17.84	12.10	6.97	18.14	14.50	8.34	21.67
Spain 2023	11.54	7.05	17.95	15.99	8.59	24.90	15.43	8.58	23.37
Spain 2024	11.77	6.94	18.10	13.15	7.33	19.59	14.62	8.44	21.55

Site–year	Tmean,PhaseA (°C)	Tmin,PhaseA (°C)	Tmax,PhaseA (°C)	Tmean,PhaseB (°C)	Tmin,PhaseB (°C)	Tmax,PhaseB (°C)	Tmean,FebMay (°C)	Tmin,FebMay (°C)	Tmax,FebMay (°C)
Türkiye 2022	9.37	5.02	14.93	17.60	10.47	25.68	11.77	5.65	18.35
Türkiye 2023	10.79	5.42	16.66	15.91	10.20	22.51	11.75	6.25	17.97
Türkiye 2024	11.23	6.96	16.50	16.92	9.70	24.21	14.22	8.01	21.08
Mean Greece	14.58	12.97	16.61	15.75	13.08	18.56	15.27	12.82	17.91
Mean Italy	12.46	9.84	16.11	14.72	10.84	18.73	13.28	9.84	17.18
Mean Morocco	15.40	10.10	23.18	18.45	11.92	26.67	18.08	11.69	26.29
Mean Spain	11.37	6.68	17.85	13.96	7.87	21.07	14.82	8.48	22.08
Mean Türkiye	10.46	5.80	16.03	16.81	10.12	24.13	12.58	6.64	19.13
Mean 2021	13.31	9.99	18.11	15.29	10.92	20.72	15.09	10.52	20.54
Mean 2022	12.22	8.07	17.77	15.62	10.21	21.88	14.66	9.37	20.80
Mean 2023	13.18	9.21	18.43	16.63	11.11	22.82	14.89	9.95	20.67
Mean 2024	12.76	9.22	17.34	15.76	10.50	21.32	15.14	10.20	20.63
Global Mean	12.90	9.12	17.98	15.88	10.72	21.78	14.93	9.99	20.66

Note: Tmean, Tmin and Tmax denote mean, minimum and maximum daily temperatures, respectively. Phase A extends from 1 October to FB50; Phase B extends from FB50 to FF50; and FebMay denotes the fixed period from 1 February to 31 May.

Table S4. Descriptive phenological statistics by germplasm-bank environment and year.

GB environment	Year	Phenological stage	n	Mean date, DOY (date)	Median date, DOY (date)	Earliest date, DOY (date)	Latest date, DOY (date)	Duration (days)	CV (%)	Mean temperature during stage (°C)
Spain	2021	FB	154	64.0 (05 Mar)	64.0 (05 Mar)	49.0 (18 Feb)	73.0 (14 Mar)	24.0	6.93%	11.2°C
		FF	180	114.2 (24 Apr)	114.0 (24 Apr)	104.0 (14 Apr)	123.0 (03 May)	19.0	2.16%	15.1°C
Spain	2022	FB	65	58.0 (27 Feb)	58.5 (27 Feb)	46.0 (15 Feb)	75.0 (16 Mar)	29.0	9.35%	11.1°C
		FF	79	118.1 (28 Apr)	118.0 (28 Apr)	108.0 (18 Apr)	128.5 (08 May)	20.5	3.12%	15.8°C
Spain	2023	FB	339	70.1 (11 Mar)	71.0 (12 Mar)	49.0 (18 Feb)	94.0 (04 Apr)	45.0	9.71%	12.7°C
		FF	377	109.0 (19 Apr)	109.0 (19 Apr)	99.5 (10 Apr)	116.0 (26 Apr)	16.5	2.59%	18.7°C
Spain	2024	FB	377	55.7 (25 Feb)	56.0 (25 Feb)	40.5 (09 Feb)	65.0 (05 Mar)	24.5	8.79%	10.4°C
		FF	424	108.3 (17 Apr)	108.5 (17 Apr)	100.0 (09 Apr)	118.0 (27 Apr)	18.0	2.03%	16.4°C
Morocco	2021	FB	90	76.0 (17 Mar)	75.2 (16 Mar)	63.0 (04 Mar)	92.2 (02 Apr)	29.2	7.21%	15.3°C
		FF	196	115.4 (25 Apr)	114.3 (24 Apr)	102.0 (12 Apr)	134.7 (15 May)	32.7	4.61%	18.3°C
Morocco	2022	FB	217	84.9 (26 Mar)	83.0 (24 Mar)	52.0 (21 Feb)	114.0 (24 Apr)	62.0	18.36%	15.5°C
		FF	65	124.2 (04 May)	124.0 (04 May)	99.5 (10 Apr)	138.5 (18 May)	39.0	7.15%	20.1°C
Morocco	2023	FB	195	81.0 (22 Mar)	82.5 (23 Mar)	72.0 (13 Mar)	87.5 (29 Mar)	15.5	4.26%	19.5°C
		FF	195	113.3 (23 Apr)	113.5 (24 Apr)	105.5 (16 Apr)	136.5 (16 May)	31.0	4.22%	24.2°C
Greece	2021	FB	40	63.2 (04 Mar)	62.0 (03 Mar)	53.0 (22 Feb)	76.0 (17 Mar)	23.0	11.66%	12.6°C
		FF	40	118.3 (28 Apr)	117.0 (27 Apr)	111.0 (21 Apr)	129.0 (09 May)	18.0	4.51%	19.5°C
Greece	2022	FB	188	85.1 (26 Mar)	90.0 (31 Mar)	51.5 (21 Feb)	109.0 (19 Apr)	57.5	14.32%	12.2°C
		FF	196	131.4 (11 May)	133.5 (14 May)	114.5 (24 Apr)	140.5 (20 May)	26.0	4.28%	17.9°C
Greece	2023	FB	89	81.1 (22 Mar)	82.0 (23 Mar)	48.5 (17 Feb)	110.0 (20 Apr)	61.5	17.33%	14.1°C
		FF	89	126.8 (07 May)	128.0 (08 May)	110.5 (20 Apr)	138.5 (18 May)	28.0	4.56%	17.3°C
Greece	2024	FB	89	75.0 (15 Mar)	77.5 (18 Mar)	52.0 (21 Feb)	90.5 (30 Mar)	38.5	12.26%	14.5°C
		FF	118	110.3 (19 Apr)	111.5 (21 Apr)	92.5 (01 Apr)	120.0 (29 Apr)	27.5	7.42%	17.7°C
Italy	2021	FB	133	89.1 (30 Mar)	90.0 (31 Mar)	70.0 (11 Mar)	102.0 (12 Apr)	32.0	7.08%	10.9°C
		FF	135	139.8 (20 May)	140.0 (20 May)	131.0 (11 May)	147.0 (27 May)	16.0	2.08%	18.6°C

GB environment	Year	Phenological stage	n	Mean date, DOY (date)	Median date, DOY (date)	Earliest date, DOY (date)	Latest date, DOY (date)	Duration (days)	CV (%)	Mean temperature during stage (°C)
Italy	2023	FB	150	74.9 (16 Mar)	76.0 (17 Mar)	54.3 (23 Feb)	104.0 (14 Apr)	49.7	12.11%	12.3°C
		FF	148	132.7 (13 May)	132.5 (12 May)	119.0 (29 Apr)	144.0 (24 May)	25.0	3.22%	17.2°C
Italy	2024	FB	0	—	—	—	—	—	—	—
		FF	97	127.3 (06 May)	125.0 (04 May)	117.0 (26 Apr)	137.5 (17 May)	20.5	3.92%	18.1°C
Türkiye	2022	FB	137	104.1 (14 Apr)	101.0 (11 Apr)	97.0 (07 Apr)	125.0 (05 May)	28.0	5.52%	14.7°C
		FF	147	144.5 (24 May)	145.0 (25 May)	143.0 (23 May)	149.0 (29 May)	6.0	0.92%	26.0°C
Türkiye	2023	FB	88	99.0 (09 Apr)	99.0 (09 Apr)	82.0 (23 Mar)	113.0 (23 Apr)	31.0	8.69%	12.4°C
		FF	63	150.7 (31 May)	152.0 (01 Jun)	149.0 (29 May)	156.0 (05 Jun)	7.0	1.26%	19.9°C
Türkiye	2024	FB	164	81.8 (22 Mar)	78.0 (18 Mar)	63.0 (03 Mar)	116.0 (25 Apr)	53.0	10.53%	13.7°C
		FF	161	129.4 (08 May)	130.0 (09 May)	120.0 (29 Apr)	141.0 (20 May)	21.0	2.36%	18.7°C
Spain	Pooled	FB	935	61.9 (03 Mar)	62.4 (03 Mar)	46.1 (15 Feb)	76.8 (18 Mar)	30.6	8.69%	11.4°C
		FF	1060	112.4 (22 Apr)	112.4 (22 Apr)	102.9 (13 Apr)	121.4 (01 May)	18.5	2.47%	16.5°C
Morocco	Pooled	FB	502	80.6 (22 Mar)	80.2 (21 Mar)	62.3 (03 Mar)	97.9 (08 Apr)	35.6	9.94%	16.8°C
		FF	456	117.6 (28 Apr)	117.3 (27 Apr)	102.3 (12 Apr)	136.6 (17 May)	34.2	5.32%	20.9°C
Greece	Pooled	FB	406	76.1 (17 Mar)	77.9 (19 Mar)	51.2 (20 Feb)	96.4 (06 Apr)	45.1	13.89%	13.3°C
		FF	443	121.7 (02 May)	122.5 (02 May)	107.1 (17 Apr)	132.0 (12 May)	24.9	5.19%	18.1°C
Italy	Pooled	FB	283	82.0 (23 Mar)	83.0 (24 Mar)	62.2 (03 Mar)	103.0 (13 Apr)	40.8	9.60%	11.6°C
		FF	380	133.3 (13 May)	132.5 (12 May)	122.3 (02 May)	142.8 (23 May)	20.5	3.07%	17.9°C
Türkiye	Pooled	FB	389	94.9 (05 Apr)	92.7 (03 Apr)	80.7 (22 Mar)	118.0 (28 Apr)	37.3	8.25%	13.6°C
		FF	371	141.5 (22 May)	142.3 (22 May)	137.3 (17 May)	148.7 (29 May)	11.3	1.51%	21.5°C
All GBs	2021	FB	417	73.1 (14 Mar)	72.8 (14 Mar)	58.8 (28 Feb)	85.8 (27 Mar)	27.1	8.22%	12.5°C
		FF	551	121.9 (02 May)	121.3 (01 May)	112.0 (22 Apr)	133.4 (13 May)	21.4	3.34%	17.9°C
All GBs	2022	FB	607	83.0 (24 Mar)	83.1 (24 Mar)	61.6 (03 Mar)	105.8 (16 Apr)	44.1	11.88%	13.4°C
		FF	487	129.5 (10 May)	130.1 (10 May)	116.2 (26 Apr)	139.1 (19 May)	22.9	3.87%	20.0°C

GB environment	Year	Phenological stage	n	Mean date, DOY (date)	Median date, DOY (date)	Earliest date, DOY (date)	Latest date, DOY (date)	Duration (days)	CV (%)	Mean temperature during stage (°C)
All GBs	2023	FB	861	81.2 (22 Mar)	82.1 (23 Mar)	61.2 (02 Mar)	101.7 (12 Apr)	40.5	10.42%	14.2°C
		FF	872	126.5 (07 May)	127.0 (07 May)	116.7 (27 Apr)	138.2 (18 May)	21.5	3.17%	19.5°C
All GBs	2024	FB	630	70.8 (12 Mar)	70.5 (11 Mar)	51.8 (21 Feb)	90.5 (31 Mar)	38.7	10.52%	12.9°C
		FF	800	118.8 (29 Apr)	118.8 (29 Apr)	107.4 (17 Apr)	129.1 (09 May)	21.8	3.93%	17.7°C
All GBs	Pooled	FB	2515	77.7 (19 Mar)	77.9 (19 Mar)	58.9 (28 Feb)	96.6 (07 Apr)	37.7	10.26%	13.3°C
		FF	2710	124.3 (04 May)	124.5 (04 May)	113.3 (23 Apr)	135.2 (15 May)	21.9	3.55%	18.8°C

Note: GB, germplasm bank; FB, Full Budburst; FF, Full Flowering; DOY, Day of the Year; n, number of observations; and CV, coefficient of variation. Period is the collection-level interval from the earliest to the latest observed date. Pooled rows summarise each GB, each year across GBs, or the complete network.

Table S5. Phenological timing and Tukey HSD groupings by germplasm-bank environment and year.

Country	Phenological Stage	n	Mean DOY	Median DOY (date)	CV (%)	Tukey HSD group
**GB environment (pooled years)**						
Spain	FB	935	62.4	62.0 (Mar 03)	13.7%	a
	FF	1060	110.3	109.0 (Apr 19)	3.6%	a
Morocco	FB	502	81.7	81.0 (Mar 22)	13.7%	bc
	FF	456	115.8	113.5 (Apr 23)	5.8%	b
Greece	FB	406	79.8	82.5 (Mar 23)	16.8%	b
	FF	443	123.7	125.0 (May 05)	8.8%	c
Italy	FB	283	81.6	82.0 (Mar 23)	13.0%	c
	FF	380	133.9	134.0 (May 14)	4.7%	d
Türkiye	FB	389	93.5	97.0 (Apr 07)	13.7%	d
	FF	371	139.0	143.0 (May 23)	6.5%	e
**Year (pooled GBs)**						
2024	FB	630	65.2	62.0 (Mar 03)	20.9%	a
	FF	800	115.1	110.0 (Apr 20)	8.9%	a
2021	FB	417	74.5	71.8 (Mar 12)	16.6%	c

Country	Phenological Stage	n	Mean DOY	Median DOY (date)	CV (%)	Tukey HSD group
	FF	551	121.2	116.0 (Apr 26)	9.4%	c
2023	FB	861	77.5	77.0 (Mar 18)	15.1%	b
	FF	872	118.8	113.5 (Apr 23)	11.2%	b
2022	FB	607	86.4	92.5 (Apr 02)	20.1%	d
	FF	487	132.2	133.5 (May 13)	8.0%	d

Note: GB, germplasm bank; FB, Full Budburst; FF, Full Flowering; DOY, Day of the Year; n, number of observations; CV, coefficient of variation; and HSD, honestly significant difference.

Table S6. Tukey HSD pairwise comparisons among germplasm-bank environments, pooled across years.

Comparison (group 2 – group 1)	Phenological Stage	Mean difference (days)	Lower 95% CI (days)	Upper 95% CI (days)	Adjusted p
Greece vs Italy	FB	1.71	-0.59	4.02	0.1617
	FF	10.18	8.83	11.54	<0.0001
Greece vs Morocco	FB	1.90	-0.09	3.89	0.0367
	FF	-7.92	-9.21	-6.63	<0.0001
Greece vs Spain	FB	-17.41	-19.18	-15.64	<0.0001
	FF	-13.39	-14.49	-12.30	<0.0001
Greece vs Türkiye	FB	13.68	11.57	15.80	<0.0001
	FF	15.31	13.95	16.67	<0.0001
Italy vs Morocco	FB	0.19	-2.03	2.40	0.9996
	FF	-18.10	-19.44	-16.76	<0.0001
Italy vs Spain	FB	-19.12	-21.14	-17.10	<0.0001
	FF	-23.58	-24.73	-22.42	<0.0001
Italy vs Türkiye	FB	11.97	9.64	14.30	<0.0001
	FF	5.12	3.71	6.53	<0.0001
Morocco vs Spain	FB	-19.31	-20.96	-17.66	<0.0001
	FF	-5.48	-6.56	-4.39	<0.0001
Morocco vs Türkiye	FB	11.78	9.77	13.79	<0.0001
	FF	23.22	21.87	24.57	<0.0001
Spain vs Türkiye	FB	31.09	29.29	32.89	<0.0001
	FF	28.70	27.53	29.87	<0.0001

Note: FB, Full Budburst; FF, Full Flowering; CI, confidence interval; and p, multiplicity-adjusted probability value. Differences are group 2 minus group 1; a positive value indicates that the second-named environment occurred later.

Table S7. Tukey HSD pairwise comparisons among years, pooled across germplasm-bank environments.

Comparison (group 2 – group 1)	Phenological Stage	Mean difference (days)	Lower 95% CI (days)	Upper 95% CI (days)	Adjusted p
2021 vs 2022	FB	11.86	9.60	14.13	<0.0001
	FF	11.01	9.15	12.86	<0.0001
2021 vs 2023	FB	2.95	0.83	5.07	0.0013
	FF	-2.38	-4.00	-0.76	0.0004
2021 vs 2024	FB	-9.29	-11.54	-7.05	<0.0001
	FF	-6.07	-7.72	-4.42	<0.0001
2022 vs 2023	FB	-8.92	-10.80	-7.03	<0.0001
	FF	-13.39	-15.07	-11.70	<0.0001
2022 vs 2024	FB	-21.16	-23.18	-19.14	<0.0001
	FF	-17.08	-18.79	-15.36	<0.0001
2023 vs 2024	FB	-12.24	-14.10	-10.38	<0.0001
	FF	-3.69	-5.15	-2.23	<0.0001

Note: FB, Full Budburst; FF, Full Flowering; CI, confidence interval; and p, multiplicity-adjusted probability value. Differences are group 2 minus group 1; a positive value indicates that the second-named year occurred later.

Table S8. Effects of germplasm-bank environment, year and their interaction on Full Budburst and Full Flowering dates.

Phenological stage	Source of variation	Sum of squares	df	Mean square	F	p	Partial η_p^2
Full Budburst (FB)	Country	821.66	4	205.42	866.78	<0.0001	0.581
	Year	220.83	3	73.61	310.61	<0.0001	0.272
	Country × Year	158.84	8	19.85	83.78	<0.0001	0.211
	Residual	592.23	2499	0.24			
Full Flowering (FF)	Country	578.37	4	144.59	3097.19	<0.0001	0.821
	Year	121.98	3	40.66	870.91	<0.0001	0.492
	Country × Year	27.65	9	3.07	65.82	<0.0001	0.180
	Residual	125.72	2693	0.05			

Note: The two-way ANOVA used Type III sums of squares on square-root-transformed Day of the Year (DOY). FB, Full Budburst; FF, Full Flowering; df, degrees of freedom; F, F-statistic; p, probability value; and partial ηp2, partial eta-squared. Country factor correspond to the environment of one GB in each country.

Table S9. Complementary linear mixed-effects analysis of Full Budburst and Full Flowering timing across olive germplasm-bank environments and years.

Stage	Original records	Cultivar groups	Observed site-years	Cultivar ICC	Country Wald χ^2 (df)	*p*	Year Wald χ^2 (df)	*p*
FB	2,515	991	16	0.400	2027.56 (4)	<0.001	1095.08 (3)	<0.001
FF	2,709	1,144	17	0.263	8097.67 (4)	<0.001	2645.28 (3)	<0.001

Note: FB, Full Budburst; FF, Full Flowering; ICC, intraclass correlation coefficient; χ^2, Wald chi-squared statistic; and df, degrees of freedom. Cultivar ICC represents the variance attributable to cultivar identity. Country and Year Wald χ^2 values are omnibus tests of the corresponding fixed effects. CountryBank represents one germplasm-bank environment per country.
